# Supplementary material for: Severe inflammation and lineage skewing are associated with poor engraftment of engineered hematopoietic stem cells in patients with sickle cell disease
Source: Nat Commun. 2025 Apr 1;16:3137. doi: 10.1038/s41467-025-58321-4 (PMC11961595; doi:10.1038/s41467-025-58321-4)
Supplement: Supplementary file 1 — Supplementary Information [file 41467_2025_58321_MOESM1_ESM.pdf]

Supplementary information

Supplementary results

Preclinical studies

First, we optimized the transduction protocol by using enhancers such as protamine sulfate (PS), prostaglandin E2 (PGE2) and Boost A in plerixafor-mobilized HSPCs from patients with SCD 13 (**Supplementary Fig. 1a-c**). After pre-activation of the SCD HSPCs for 48 hours, the DREPAGLOBE vector (**Supplementary Fig. 1a**) and one or more transduction enhancers were added for 24 hours. The combined use of the three transduction enhancers yielded a mean vector copy number (VCN) of 1.14 in HSPCs grown in liquid culture for 14 days after transduction (**Supplementary Fig. 1b**) and high levels of expression of the therapeutic hemoglobin containing the βAS3 globin chain (HbAS3) in HSPC-derived burst-forming unit erythroid (BFU-E) colonies, as determined by HPLC (**Supplementary Fig. 1c**).

We tested the safety and efficacy of the DREPAGLOBE vector in transducing SCD patients' cells under Good Manufacturing Practice conditions (**Supplementary Fig. 1d-f**). After transduction, cells were grown either in liquid culture or in a semi-solid medium allowing the clonal growth of BFU-E and granulomonocytic (CFU-GM) progenitors (CFC assay). No differences in the clonogenic potential were observed between mock- and LV-transduced HSPCs (**Supplementary Fig. 1d).** The vector copy number (VCN) was between 0.88 and 1.19 in liquid cultures, 1.12 to 1.91 in BFU-E, and 0.66 to 1.60 in CFU-GM (**Supplementary Fig. 1e**). The Hb containing the βAS3 globin chain in BFU-E represented ~30% of the total Hb as determined by HPLC (**Supplementary Fig. 1f**).

Finally, we assessed the efficiency of this transduction protocol *in vivo* in *bona fide* SCD hematopoietic stem cells (HSCs). HSPCs transduced in Good Manufacturing Practice conditions were able to engraft in busulfan-conditioned NSG immunodeficient mice (**Supplementary Fig. 1g**) and gave rise to the different blood lineages, as evaluated by flow cytometry in the bone marrow 18-19 weeks post-transplantation (**Supplementary Fig. 1h**). Importantly, the combined use of PS, PGE2 and Boost A led to the highest VCN in repopulating human HSPCs, which ranged between 1.77 and 3.80 (**Supplementary Fig. 1i**).

Harvesting, backup cell collection and recovery of CD34+ HSPCs

Two consecutive days of cell harvesting by apheresis were necessary to obtain a sufficient number of CD34+ HSPCs to transduce. For P1, the backup was taken from the pool of the two collections (day 1 and day 2). For P2, the backup of cells had already been cryopreserved in the Drepamob clinical trial (NCT02212535). For P3, a third day of harvesting was scheduled to collect the backup cells. For P4, the back-up was taken from the cells collected on day 2 (**Supplementary Table 1)**.

The recovery of CD34+ cells after selection was variable (**Supplementary Table 1),** but in the range of previously reported values in patients with SCD[1,2] and in patients with other diseases or in healthy donors[3]. However, it is interesting to note that while we harvested the highest number of cells for the youngest patients (P1 and P4) (**Supplementary Table 1)**, the recovery was lower compared to the older patients suggesting that other factors influence the total number of HSPCs obtained after selection even in young patients. Furthermore, despite a similar cell dose in the DP, P3 and P4 received a lower dose of *bona fide* HSCs, as evaluated by multi-parameter flow cytometry (**Supplementary Table 1)**.

Supplementary materials

Transfer vector sequence

```
   1 ccattgcata cgttgtatcc atatcataat atgtacattt atattggctc atgtccaaca
  61 ttaccgccat gttgacattg attattgact agttattaat agtaatcaat tacggggtca
 121 ttagttcata gcccatatat ggagttccgc gttacataac ttacggtaaa tggcccgcct
 181 ggctgaccgc ccaacgaccc ccgcccattg acgtcaataa tgacgtatgt tcccatagta
 241 acgccaatag ggactttcca ttgacgtcaa tgggtggagt atttacggta aactgcccac
 301 ttggcagtac atcaagtgta tcatatgcca agtacgcccc ctattgacgt caatgacggt
 361 aaatggcccg cctggcatta tgcccagtac atgaccttat gggactttcc tacttggcag
 421 tacatctacg tattagtcat cgctattacc atggtgatgc ggttttggca gtacatcaat
 481 gggcgtggat agcggtttga ctcacgggga tttccaagtc tccaccccat tgacgtcaat
 541 gggagtttgt tttggcacca aaatcaacgg gactttccaa aatgtcgtaa caactccgcc
 601 ccattgacgc aaatgggcgg taggcgtgta cggtgggagg tctatataag cagagctcgt
 661 ttagtgaacc ggggtctctc tggttagacc agatctgagc ctgggagctc tctggctaac
 721 tagggaaccc actgcttaag cctcaataaa gcttgccttg agtgcttcaa gtagtgtgtg
 781 cccgtctgtt gtgtgactct ggtaactaga gatccctcag acccttttag tcagtgtgga
 841 aaatctctag cagtggcgcc cgaacaggga cttgaaagcg aaagggaaac cagaggagct
 901 ctctcgacgc aggactcggc ttgctgaagc gcgcacggca agaggcgagg ggcggcgact
 961 ggtgagtacg ccaaaaattt tgactagcgg aggctagaag gagagagatg ggtgcgagag
1021 cgtcagtatt aagcggggga gaattagatc gcgatgggaa aaaattcggt taaggccagg
1081 gggaaagaaa aaatataaat taaaacatat agtatgggca agcagggagc tagaacgatt
1141 cgcagttaat cctggcctgt tagaaacatc agaaggctgt agacaaatac tgggacagct
1201 acaaccatcc cttcagacag gatcagaaga acttagatca ttatataata cagtagcaac
1261 cctctattgt gtgcatcaaa ggatagagat aaaagacacc aaggaagctt tagacaagat
1321 agaggaagag caaaacaaaa gtaagaccac cgcacagcaa gcggccgctg atcttcagac
1381 ctggaggagg agatatgagg gacaattgga gaagtgaatt atataaatat aaagtagtaa
1441 aaattgaacc attaggagta gcacccacca aggcaaagag aagagtggtg cagagagaaa
1501 aaagagcagt gggaatagga gctttgttcc ttgggttctt gggagcagca ggaagcacta
1561 tgggcgcagc gtcaatgacg ctgacggtac aggccagaca attattgtct ggtatagtgc
1621 agcagcagaa caatttgctg agggctattg aggcgcaaca gcatctgttg caactcacag
1681 tctggggcat caagcagctc caggcaagaa tcctggctgt ggaaagatac ctaaaggatc
1741 aacagctcct ggggatttgg ggttgctctg gaaaactcat ttgcaccact gctgtgcctt
1801 ggaatgctag ttggagtaat aaatctctgg aacagatttg gaatcacacg acctggatgg
1861 agtgggacag agaaattaac aattacacaa gcttaataca ctccttaatt gaagaatcgc
1921 aaaaccagca agaaaagaat gaacaagaat tattggaatt agataaatgg gcaagtttgt
1981 ggaattggtt taacataaca aattggctgt ggtatataaa attattcata atgatagtag
2041 gaggcttggt aggtttaaga atagtttttg ctgtactttc tatagtgaat agagttaggc
2101 agggatattc accattatcg tttcagaccc acctcccaac cccgagggga cccgacaggc
2161 ccgaaggaat agaagaagaa ggtggagaga gagacagaga cagatccatt cgattagtga
2221 acggatctcg acggtatcgg ttaactttta aaagaaaagg ggggattggg gggtacagtg
2281 caggggaaag aatagtagac ataatagcaa cagacataca aactaaagaa ttacaaaaac
2341 aaattacaaa attcaaaatt ttatcggtac gtaccatgag gacagctaaa acaataagta
2401 atgtaaaata cagcatagca aaactttaac ctccaaatca agcctctact tgaatccttt
2461 tctgagggat gaataaggca taggcatcag ggctgttgc caatgtgcat tagctgtttg
2521 cagcctcacc ttctttcatg gagtttaaga tatagtgtat tttcccaagg tttgaactag
2581 ctcttcattt ctttatgttt taaatgcact gacctcccac attccctttt tagtaaaata
2641 ttcagaaata atttaaatac atcattgcaa tgaaaataaa tgttttttat taggcagaat
2701 ccagatgctc aaggcccttc ataatatccc ccagtttagt agttggactt agggaacaaa
2761 ggaaccttta atagaaattg gacagcaaga aagcgagctt agtgatactt gtgggccagg
```

2821 gcattagcca caccagccac cactttctga taggcagcct gcactggtgg ggtgaattct
2881 ttgccaaagt gatgggccag cacacagacc agcacgttgc ccaggagctg tgggaggaag
2941 ataagaggta tgaacatgat tagcaaaagg gcctagcttg gactcagaat aatccagcct
3001 tatcccaacc ataaaataaa agcagaatgg tagctggatt gtagctgcta ttagcaatat
3061 gaaacctctt acatcagtta caatttatat gcagaaatac cctgttactt ctccccttcc
3121 tatgacatga acttaaccat agaaaagaag ggaaagaaa acatcaaggg tcccatagac
3181 tcaccctgaa gttctcagga tccacgtgca gcttgtcaca gtgcagctca ctcagctggg
3241 caaaggtgcc cttgaggttg tccaggtgag ccaggccatc actaaaggca ccgagcactt
3301 tcttgccatg agccttcacc ttagggttgc ccataacagc atcaggagtg gacagatccc
3361 caaaggactc aaagaacctc tgggtccaag ggtagaccac cagcagccta agggtgggaa
3421 aatagaccaa taggcagaga gagtcagtgc ctatcagaaa cccaagagtc ttctctgtct
3481 ccacatgccc agtttctatt ggtctcctta aacctgtctt gtaaccttga taccaacctg
3541 cccagggcct caccaccaac ggcatccacg ttcaccttgt cccacagggc agtaacggca
3601 gacttctcct caggagtcag gtgcaccatg gtgtctgttt gaggttgcta gtgaacacag
3661 ttgtgtcaga agcaaatgta agcaatagat ggctctgccc tgacttttat gcccagccct
3721 ggctcctgcc ctccctgctc ctgggagtag attggccaac cctagggtgt ggctccacag
3781 ggtgaggtct aagtgatgac agccgtacct gtccttggct cttctggcac tggcttagga
3841 gttggacttc aaaccctcag ccctccctct aagatatatc tcttggcccc ataccatcag
3901 tacaaattgc tactaaaaac atcctccttt gcaagtgtat ttacacggta tcgataagct
3961 tgatatcgaa ttcctgcagc cccctttgc cacctagctg tccaggggtg ccttaaaatg
4021 gcaaacaagg tttgttttct tttcctgttt tcatgccttc ctcttccata tccttgtttc
4081 atattaatac atgtgtatag atcctaaaaa tctatacaca tgtattaata aagcctgatt
4141 ctgccgcttc taggtataga ggccacctgc aagataaata tttgattcac aataactaat
4201 cattctatgg caattgataa caacaaatat atatatatat atatatacgt atatgtgtat
4261 atatatatat atatattcag gaaataatat attctagaat atgtcacatt ctgtctcagg
4321 catccatttt ctttatgatg ccgtttgagg tggagtttta gtcaggtggt cagcttctcc
4381 tttttttgc catctgccct gtaagcatcc tgctggggac ccagatagga gtcatcactc
4441 taggctgaga acatctgggc acacacccta agcctcagca tgactcatca tgactcagca
4501 ttgctgtgct tgagccagaa ggtttgctta gaaggttaca cagaaccaga aggcgggggt
4561 ggggcactga ccccgacagg ggcctggcca gaactgctca tgcttggact atgggaggtc
4621 actaatggag acacacagaa atgtaacagg aactaaggaa aaactgaagc ttatttaatc
4681 agagatgagg atgctggaag ggatagaggg agctgagctt gtaaaaagta tagtaatcat
4741 tcagcaaatg gttttgaagc acctgctgga tgctaaacac tattttcagt gcttgaatca
4801 taaataagaa taaaacatgt atcttattcc ccacaagagt ccaagtaaaa aataacagtt
4861 aattataatg tgctctgtcc cccaggctgg agtgcagtgg cacgatctca gctcactgca
4921 acctccgcct cccgggttca agcaattctc ctgcctcagc caccctaata gctgggatta
4981 caggtgcaca ccaccatgcc aggctaattt ttgtactttt tgtagaggca gggtatcacc
5041 atgttgtcca agatggtctt gaactcctga gctccaagca gtccacccac ctcagcctcc
5101 caaagtgctg ggattacagg tgtgagacac catgcccaga ttttccatat ttaatagagg
5161 tatttatggg atgggggaaa agaatgtttc tctcactgtg gattatttta gagagtggag
5221 aatggtcaag attttttaa aaattaagaa aacataagtt ggaccttgag aaatgaaaat
5281 ttattttttt gttggaggat acccattctc tatctcccat cagggcaagc tgtaaggaac
5341 tggctaagac acagtgagac agagtgactt agtcttagag gccccactgg tacgacggtc
5401 accaagcttt cattaaaaaa agtctaacca gctgcattcg actttgactg cagcagctgg
5461 ttagaaggtt ctactggagg agggtcccag cccattgcta aattaacatc aggctctgag
5521 actggcagta tatctctaac agtggttgat gctatcttct ggaacttgcc tgctacattg
5581 agaccactga cccatacata ggaagcccat agctctgtcc tgaactgtta ggccactggt
5641 ccagagagtg tgcatctcct ttgatcctca taataaccct atgagataga cacaattatt
5701 actcttactt tatagatgat gatcctgaaa acataggagt caaggcactt gcccctagct
5761 gggggtatag ggagcagtc ccatgtagta gtagaatgaa aaatgctgct atgctgtgcc
5821 tcccccacct ttcccatgtc tgccctctac tcatggtcta tctctcctgg ctcctgggag

5881 tcatggactc cacccagcac caccaacctg acctaaccac ctatctgagc ctgccagcct

5941 ataacccatc tgggccctga tagctggtgg ccagccctga ccccacccca ccctccctgg

6001 aacctctgat agacacatct ggcacaccag ctcgcaaagt caccgtgagg gtcttgtgtt

6061 tgctgagtca aaattccttg aaatccaagt ccttagagac tcctgctccc aaatttacag

6121 tcatagactt cttcatggct gtctccttta tccacagaat gattcctttg cttcattgcc

6181 ccatccatct gatcctcctc atcagtgcag cacagggccc atgagcagta gctgcagagt

6241 ctcacatagg tctggcactg cctctgacat gtccgacctt aggcaaatgc ttgactcttc

6301 tgagctcagt cttgtcatgg caaaataaag ataataatag tgttttttta tggagttagc

6361 gtgaggatgg aaaacaatag caaaattgat tagactataa aaggtctcaa caaatagtag

6421 tagattttat catccattaa tccttccctc tcctctctta ctcatcccat cacgtatgcc

6481 tcttaatttt cccttaccta taataagagt tattcctctt attatattct tcttatagtg

6541 attctggata ttaaagtggg aatgaggggc aggccactaa cgaagaagat gtttctcaaa

6601 gaagcggggg atccactagt tctagagcgg ccaaatggcg gccgtacctt taagaccaat

6661 gacttacaag gcagctgtag atcttagcca ctttttaaaa gaaaaggggg gactggaagg

6721 gctaattcac tcccaacgaa gacaagatct gctttttgct tgtactgggt ctctctggtt

6781 agaccagatc tgagcctggg agctctctgg ctaactaggg aacccactgc ttaagcctca

6841 ataaagcttg ccttgagtgc ttcaagtagt gtgtgcccgt ctgttgtgtg actctggtaa

6901 ctagagatcc ctcagaccct tttagtcagt gtggaaaatc tctagcagta gtagttcatg

6961 tcatcttatt attcagtatt tataacttgc aaagaaatga atatcagaga gtgagaggaa

7021 cttgtttatt gcagcttata atggttacaa ataaagcaat agcatcacaa atttcacaaa

7081 taaagcattt ttttcactgc attctagttg tggtttgtcc aaactcatca atgtatctta

7141 tcatgtctgg ctctagctat cccgccccta actccgccca tcccgcccct aactccgccc

7201 agttccgccc attctccgcc ccatggctga ctaatttttt ttatttatgc agaggccgag

7261 gccgcctcgg cctctgagct attccagaag tagtgaggag gcttttttgg aggcctaggg

7321 acgtacccaa ttcgccctat agtgagtcgt attacgcgcg ctcactggcc gtcgttttac

7381 aacgtcgtga ctgggaaaac cctggcgtta cccaacttaa tcgccttgca gcacatcccc

7441 ctttcgccag ctggcgtaat agcgaagagg cccgcaccga tcgcccttcc caacagttgc

7501 cctagggacg tacccaattc gccctatagt gagtcgtatt acgcgcgctc actggccgtc

7561 gttttacaac gtcgtgactg ggaaaaccct ggcgttaccc aacttaatcg ccttgcagca

7621 catccccctt tcgccagctg gcgtaatagc gaagaggccc gcaccgatcg cccttcccaa

7681 cagttgcgca gcctgaatgg cgaatgggac gcgccctgta gcggcgcatt aagcgcggcg

7741 ggtgtggtgg ttacgcgcag cgtgaccgct acacttgcca gcgccctagc gcccgctcct

7801 ttcgctttct tcccttcctt tctcgccacg ttcgccggct ttccccgtca agctctaaat

7861 cgggggctcc ctttagggtt ccgatttagt gctttacggc acctcgaccc caaaaaactt

7921 gattagggtg atggttcacg tagtgggcca tcgccctgat agacggtttt tcgccctttg

7981 acgttggagt ccacgttctt taatagtgga ctcttgttcc aaactggaac aacactcaac

8041 cctatctcgg tctattcttt tgatttataa gggattttgc cgatttcggc ctattggtta

8101 aaaaatgagc tgatttaaca aaaatttaac gcgaatttta acaaaatatt aacgcttaca

8161 atttaggtgg cacttttcgg ggaaatgtgc gcggaacccc tatttgttta tttttctaaa

8221 tacattcaaa tatgtatccg ctcatgagac aataaccctg ataaatgctt caataatagc

8281 acctagatca agagacagga tgaggatcgt ttcgcatgat tgaacaagat ggattgcacg

8341 caggttctcc ggccgcttgg gtggagaggc tattcggcta tgactgggca caacagacaa

8401 tcggctgctc tgatgccgcc gtgttccggc tgtcagcgca ggggcgcccg gttctttttg

8461 tcaagaccga cctgtccggt gccctgaatg aactgcaaga cgaggcagcg cggctatcgt

8521 ggctggccac gacgggcgtt ccttgcgcag ctgtgctcga cgttgtcact gaagcgggaa

8581 gggactggct gctattgggc gaagtgccgg ggcaggatct cctgtcatct caccttgctc

8641 ctgccgagaa agtatccatc atggctgatg caatgcggcg gctgcatacg cttgatccgg

8701 ctacctgccc attcgaccac caagcgaaac atcgcatcga gcgagcacgt actcggatgg

8761 aagccggtct tgtcgatcag gatgatctgg acgaagagca tcaggggctc gcgccagccg

8821 aactgttcgc caggctcaag gcgagcatgc ccgacggcga ggatctcgtc gtgacccatg

8881 gcgatgcctg cttgccgaat atcatggtgg aaaatggccg cttttctgga ttcatcgact

```
 8941 gtggccggct gggtgtggcg gaccgctatc aggacatagc gttggctacc cgtgatattg
 9001 ctgaagagct tggcggcgaa tgggctgacc gcttcctcgt gctttacggt atcgccgctc
 9061 ccgattcgca gcgcatcgcc ttctatcgcc ttcttgacga gttcttctga attattaacg
 9121 cttacaattt cctgatgcgg tattttctcc ttacgcatct gtgcggtatt tcacaccgca
 9181 tcaggtggca cttttcgggg aaatgtgcgc ggaaccccta tttgtttatt tttctaaata
 9241 cattcaaata tgtatccgct catgaccaaa atcccttaac gtgagttttc gttccactga
 9301 gcgtcagacc ccgtagaaaa gatcaaagga tcttcttgag atcctttttt tctgcgcgta
 9361 atctgctgct tgcaaacaaa aaaaccaccg ctaccagcgg tggtttgttt gccggatcaa
 9421 gagctaccaa ctctttttcc gaaggtaact ggcttcagca gagcgcagat accaaatact
 9481 gttcttctag tgtagccgta gttaggccac cacttcaaga actctgtagc accgcctaca
 9541 tacctcgctc tgctaatcct gttaccagtg gctgctgcca gtggcgataa gtcgtgtctt
 9601 accgggttgg actcaagacg atagttaccg gataaggcgc agcggtcggg ctgaacgggg
 9661 ggttcgtgca cacagcccag cttggagcga acgacctaca ccgaactgag atacctacag
 9721 cgtgagctat gagaaagcgc cacgcttccc gaagggagaa aggcggacag gtatccggta
 9781 agcggcaggg tcggaacagg agagcgcacg agggagcttc cagggggaaa cgcctggtat
 9841 ctttatagtc ctgtcgggtt tcgccacctc tgacttgagc gtcgattttt gtgatgctcg
 9901 tcaggggggc ggagcctatg gaaaaacgcc agcaacgcgg cctttttacg gttcctggcc
 9961 ttttgctggc cttttgctca catgttcttt cctgcgttat cccctgattc tgtggataac
10021 cgtattaccg cctttgagtg agctgatacc gctcgccgca gccgaacgac cgagcgcagc
10081 gagtcagtga gcgaggaagc ggaagagcgc ccaatacgca aaccgcctct ccccgcgcgt
10141 tggccgattc attaatgcag ctggcacgac aggtttcccg actggaaagc gggcagtgag
10201 cgcaacgcaa ttaatgtgag ttagctcact cattaggcac cccaggcttt acactttatg
10261 cttccggctc gtatgttgtg tggaattgtg agcggataac aatttcacac aggaaacagc
10321 tatgaccatg attacgccaa gcgcgcaatt aaccctcact aaagggaaca aaagctggag
10381 ctgcaagctt gg
```

Supplementary figure legends

Supplementary Fig. 1 Preclinical studies to optimize transduction of SCD HSPCs in Good Manufacturing Practice conditions. (**a**) Experimental scheme created in BioRender. Lab, M. (2025). HSPCs from SCD patients were transduced with DREPAGLOBE LV and subjected to liquid culture and CFC assay, or transplanted in NGS mice. Schematic representation of β-AS3 HS4 and β-AS3 lentiviral vectors. Δ, deleted HIV-1 U3 region; SD and SA, HIV Splicing Donor and Acceptor sites; γ, HIV-1 packaging signal; RRE, HIV-1 Rev Responsive Element; Ex, exons of the human *HBB*; βp, promoter of *HBB*; HS2, 3 and 4: DNase I hypersensitive site 2, 3 and 4 of human *HBB* LCR; red arrows indicate the mutations introduced in exon 1 (generating amino acid substitutions G16D and E22A) and exon 2 (generating amino acid substitution T87Q). (**b**) VCN in liquid cultures of SCD HSPCs after transduction in the presence of protamine sulfate (PS), PGE2 and Boost A. Data are expressed as mean ± SD (n=3 biologically independent experiments, 2 donors). (**c**) CE-HPLC chromatograms of hemoglobin tetramers in SCD HSPCs-derived BFU-E. (**d**) CFC frequency for control and transduced samples. Data are expressed as mean ± SD (n=3 biologically independent experiments, 2 donors). (**e**) VCN in liquid cultures, SCD HSPC-derived BFU-E and CFU-GM after transduction in Good Manufacturing Practice conditions. Data are expressed as mean ± SD (n=3 biologically independent experiments, 2 donors). (**f**) Percentage of HbAS3 n SCD HSPC-derived BFU-E after transduction in Good Manufacturing Practice conditions, as assessed by CE-HPLC. Data are expressed as mean ± SD (n=3 biologically independent experiments, 2 donors). (**g**) Engraftment of human cells in NSG mice transplanted with control (mock-transduced) and transduced SCD HSPCs (n=4 mice per group) 18 to 19 weeks post-transplantation. Engraftment is represented as the percentage of human $CD45^+$ cells in the total murine and human $CD45^+$ cell population, in BM, or as total number of human $CD45^+$ cells in the BM. Each data point represents an individual mouse. Data are expressed as mean ± SEM. No statistical differences were observed between control and edited samples for the other comparisons (Mann-Whitney test). (**h**) Human hematopoietic cell reconstitution in NSG mice transplanted with control and transduced HSPCs. We plotted the frequency of human T (CD3) and B (CD19) lymphoid, myeloid (CD14 and CD15), and erythroid (CD36 and CD71) cells in BM in mice transplanted with control and transduced HSPCs (n=4 mice per group). Each data point represents an individual mouse. Data are expressed as mean ± SD. (**i**) VCN on sorted human CD45. Each data point represents an individual mouse. Significant differences were observed between TD+PGE2 and TD+PGE2+BoostA (*p<0.05) or no statistical differences were observed between control and edited samples for the other comparisons (Mann-Whitney test). Source data are provided as a Source Data file.

Supplementary Fig. 2 List of adverse events (AEs) and severe adverse events (SAEs) and integration sites analysis in PBMCs. (**a**) List of ≥ grade 3 AEs and SAEs (except VOC or ACS) occurred after neutrophil engraftment (**b**) Diversity of unique integration sites (IS) quantified for the 4 SCD patients using the Shannon diversity index. (**c**) Relative clonal abundance for each of the four SCD patients. Clone size was quantified using the Sonic Abundance methods56. Each of the most abundant clones is indicated by a different color; the nearest gene to each abundant integration site is named at the right. White shows the proportion contributed to all other ISs. The total number of ISs is indicated at the top of each column. ~ indicates oncogenes. (**d**) IS richness for the 4 SCD patients estimated using Chao1 estimator, which provides a lower bound on the reconstructed population size[4].

Supplementary Fig. 3 Gene marking and expression of hemoglobins after GT. (**a**) VCN kinetics in mature blood cell sub-populations of SCD patients. PBMC, peripheral blood mononuclear cells. (**b**) VCN kinetics in granulocyte-macrophage colony-forming units (CFU-GM) and erythroid burst-forming units (BFU-E). (**c**) Distribution of HbF-expressing RBCs in SCD patients' blood at 18 months by flow cytometry using an antibody that recognizes HbF. (**d**) Hb concentrations assessed by HPLC in patients' blood. HU, hydroxyurea. (**e**) Mean

amount of HbAS3 per CD71+ reticulocyte, expressed in picograms, in HbAS3+ cells (calculated using the formula: HbAS3%-assessed by HPLC in sorted CD71+ x MCH/HbAS3+-CD71+%-assessed by flow cytometry). Source data are provided as a Source Data file.

Supplementary Fig. 4 Correction of erythropoiesis in GT-treated patients and functional evaluation of RBC parameters, hemolysis markers, inflammation and liver and heart overload. (**a**) Photomicrographs of P2 erythroid precursors before and after GT. (**b**) *In vitro* sickling of RBCs at 5% O_2. Representative images of RBCs under 5% O_2 from P1 and P2 and a control non-treated SCD patient are shown (top panels). We plotted the percentage of *in vitro* sickled RBCs at 5% O_2 from P1 and P2 and a control non-treated SCD patient (n= 4 different fields; bottom panel). (**c**) Photomicrographs of P2 RBCs before and after GT. (**d**) RBC deformability under normoxia and increasing osmotic gradient from 60 to 500 mOsm/kg, measured at constant shear stress of 30 Pa, for P1 and P2. Maximum deformability for P1 and P2 was similar to HD and higher than SS patients. Due to massive blood exchange transfusion, the D0 curve is similar to the healthy donors' curve. Both P1 and P2 exhibited improved overall hydration after GT as compared to SS patients (O hyper points). The gray area represents the interquartile range (IQR) measured in 23 untransfused SS patients. (**e**) RBC deformability under oxygen gradient, measured at month 18 at constant shear stress of 30 Pa. The gray area represents the interquartile range (IQR) measured in n = 19 untransfused SS patients. (**f**) Frequency of dense RBCs defined as RBCs with density ≥1.110 mg/mL over time for P1 and P2. The gray area represents the IQR dense RBC% measured in 32 SS patients. (**g**) Dissociation and association of O2 curves with RBCs from P1 at month 18 and from P2 at month 24. (**h**) Evolution of hemolysis markers after GT in the blood of treated SCD patients. Gray and brown areas show SS and HD normal ranges, respectively. (**i**) Evolution of CRP levels in the blood of treated SCD patients. Brown area shows HD normal ranges. (**j-k**) Non-invasive in vivo quantification of tissue iron levels in SCD patients by 1.5 Tesla magnetic resonance imaging (MRI) in liver (R2*/T2* relaxometry) (**j**) and heart (T2*) (**k**). Liver iron levels were in the normal range before and after GT for P3 who was the only patient compliant with the iron-chelator treatment. Brown areas show HD normal ranges. Source data are provided as a Source Data file.

Supplementary Fig. 5 Multiparametric spectral flow cytometry analysis of HSPCs. (**a**) t-SNE representation of CD34+Lin- HSPCs by individuals using the OMIQ software and depicting the various HSPC subpopulations as shown in the color code on the right. For each patient, subpopulations showing frequency variation, in patients compared with HD, are highlighted with a color name label (**b**) CMP, GMP and DCP frequencies in total HSPCs. (**c**) Percentage of CFU-GM and BFU-E obtained by plating 500 HSPCs in Methylcellulose colony forming unit (CFU) assay. (**d**) B and NK progenitor frequencies among total HSPCs. (**e**) MEP frequency among total HSPCs. (**f**) Percentages of MEP expressing the combination of the erythroid CD71 and megakaryocytic CD41 marker. (**g**) t-SNE representation of CD71, CD41 and CD110 expression on CD34+Lin- HSPCs by individual using the OMIQ software. (**h-i**) Flow cytometry plots showing CD71 and CD41 expression in HD2 and P3 on MEPs and HSCs. Source data are provided as a Source Data file.

Supplementary Fig. 6 Mixed signature identification in patient P3 using Cell-ID method. (**a**) Bar plots showing the percentages of the different subpopulations in HSC-enriched, MPP, MLP, BcellP, ImP1, ImP2, EryP, EoBasMastP and NA in each individual (related to Fig. 4). (**b**) Repartition of the number of matches with the reference Velten signature for each individual. The frequency of cells matching 2 signatures or more, a single signature or not significantly matching any is shown respectively in black, gray and white. (**c**) UMAP representation of all P3 cells that match significantly with MkP signature (Cell Match, $p<0.05$). (**d**) UMAP representation of all P3 cells with the top p-value for MkP label (Cell ID, $p<0.05$). (**e**) UpSet plots showing the number of cells significantly matching with one or more cell types: All HSC (a cell population including the most immature HSCs and the HSC-enriched subpopulations), MPP, MonoDCP, NeutroP, MEP and MkP signatures and the other subpopulation (i.e. the 7

other HSPC cell type signatures) ($p<0.05$). The top 12 lineage combinations are shown per individual. The arrows indicate cells identified as simultaneously displaying signatures for MkP and All HSC. P3 presented a larger number of MkP cells with mixed signatures compared with other patients and HDs. The dotted box highlights the predominant cell types in P3 (MkP). (related to Fig. 5a). (**f**) Genes belonging to IFN alpha and gamma pathways and differentially expressed between patients' and HDs' HSCs. Purple, genes belonging to the IFN gamma signature; Turquoise, genes belonging to the IFN alpha signature; Black, genes present in both pathways. (**g**) Genes belonging to TNF alpha pathway and differentially expressed between patients' and HDs' HSCs.

Supplementary Fig. 7 Inflammatory signatures from 4 β-thalassemia patients treated by gene therapy. (**a**) VCN kinetics in neutrophils of four transfusion-dependent thalassemia (TDT) patients. (**b**) Frequency of HSCs in the DP using flow cytometry. (**c**) Number of infused corrected HSCs/kg. (**d**) Top 10 enriched Hallmark genesets in TDT vs HDs HSPCs. RNA-seq data were analyzed using DESeq2. We performed a hypergeometric test with MSigDB on the DEG using Hallmark genesets ($Log_2FC>1.2$, $FDR<0.05$). (**e**) Representation and quantification of modules of genesets activity with the ROMA tool in individual HSPC samples using selected hallmark genesets. Red arrows indicate inflammatory genesets. (**f**) Unsupervised analysis of 43,343 HSPCs and 15,831 genes from 2 HDs and from 4 patients with TDT, represented as two-dimensional UMAP plots. Each individual cell in our dataset was annotated using the Cell-ID method and reference BM HSPC signatures[5]. (**g**) Top 10 pathways (in terms of p-value, identified using a hypergeometric test, MSigDB and hallmark genesets) among the 1,118 DEGs identified with the MAST tool in TDT HSCs (n=4) vs. HD HSCs (n=2). In each pathway, genes that are upregulated in TDT (relative to HDs) are shown in red, and those that are downregulated in TDT are shown in blue. The false discovery rate ($-log_{10}$(adjusted p-value)) is shown for each pathway. The numbers of upregulated and downregulated genes in each pathway are also shown. (**h-i**) For each HD (n=2) and TDT patient (n=4), a significant TNFa pathway enrichment score ($p<0.01$) is shown on HSPCs, using UMAP plots (h), and on HSCs, using boxplot. Dotted lines represent the significant threshold $-log_{10}$(p-value) (i). (**j-k**) For each HD (n=2) and TDT patient (n=4), a significant IFN gamma response pathway enrichment score ($p<0.01$) is shown on HSPCs, using UMAP plots (j) and on HSCs, using boxplot. Dotted lines represent the significant threshold $-log_{10}$(p-value) (k). Source data are provided as a Source Data file.

Supplementary Fig. 8 Identification of increased aging signature in HSCs of patients presenting defective long-term engraftment. (**a-b**) Aged vs. Young HSC enrichment score ($p<0.01$) is shown in HSPCs, using UMAP plots in SCD (a) and TDT (b) patients respectively compared to HDs. (**c-d**) Aged vs. Young HSC enrichment score is shown in HSCs and HSC-enriched populations, using boxplot. Dotted lines represent the significant threshold $-log_{10}$(p-value). (**e**) Pearson correlation between the median of Aged vs. Young HSC score in HSCs and the individual age. The table on the right reported rho correlation value (r) and p-value considering HD individuals alone, or all individuals (HD + patients).

Supplementary Information References

1. Lagresle-Peyrou, C. *et al.* Plerixafor enables safe, rapid, efficient mobilization of hematopoietic stem cells in sickle cell disease patients after exchange transfusion. *Haematologica* 103, 778–786 (2018).
2. Esrick, E. B. *et al.* Successful hematopoietic stem cell mobilization and apheresis collection using plerixafor alone in sickle cell patients. *Blood Adv* 2, 2505–2512 (2018).
3. Calmels, B. *et al.* [CD34+ cell selection methods, quality controls and expected results: Guidelines from the Francophone Society of Bone Marrow Transplantation and Cellular Therapy (SFGM-TC)]. *Bull Cancer* 107, S185–S192 (2020).
4. Bushman, F. D., Cantu, A., Everett, J., Sabatino, D. & Berry, C. Challenges in estimating numbers of vectors integrated in gene-modified cells using DNA sequence information. *Mol Ther* 29, 3328–3331 (2021).
5. Sobrino, S. *et al.* Severe hematopoietic stem cell inflammation compromises chronic granulomatous disease gene therapy. *Cell Rep Med* 4, (2023).

Supplementary Table 1. Clinical features of the patients treated by gene therapy and characteristics of the drug product

	P1	P2	P3	P4
**Patients**				
**Sex**	M	M	M	F
**Follow up post GT (months)**	48	42	36	24
**Genotype**	β^S/β^S with single 3.7-kb α-globin gene deletion	β^S/β^S	β^S/β^S , bi-allelic rs7482144 SNP in the *HBG2* γ-globin promoter	β^S/β^S
**Treatment process**				
**HSPCs (x10^6/kg) before selection**	13.25	8.7	6	17.11
**HSPCs (x10^6/kg) before selection after removing the back-up**	11.03	NA*	NA*	15.03
**HSPCs (x10^6/kg) seeded in culture at day 0 (recovery)**	7.22 (65%)	6.27 (72%)	5.73 (96%)	8.36 (55%)
**Busulfan AUC (daily average, µM*min)**	4.062	4.087	4.867	4.833
**Day to neutrophil engraftment**	14	14	19	17
**Day to platelet engraftment**	63	19	-**	20
**Day of hospitalization from conditioning to discharge**	31	32	37	44
**Drug product**				
**VCN in drug product (day 3)**	0.75	1.17	0.91	1.05
**VCN in neutrophils at the last FU**	0.44 (M48)	0.94 (M42)	0.25 (M36)	0.19 (M24)
**HSPCs dose (x10^6/kg)**	8.10	6.62	5.97	6.39
**HSCs dose (x10^6/kg)**	0.05	0.04	0.02	0.02

*NA, not applicable. For P2 unselected CD34+ cells/kg were cryopreserved as a back-up in the NCT02212535 clinical trial. For P3 a third day of harvesting was scheduled to collect CD34+ cells/kg that were cryopreserved as a back-up.
** Platelet numbers never went below 50,000/10^9/L

HSPCs: hematopoietic stem and progenitor cells, GT: gene therapy, VCN: vector copy number

Supplementary Table 2. Biological parameters and transfusion regimen before infusion of the DP

		Normal Range		P1	P2	P3	P4
Biological parameters	HbS% prior hypertransfusion	NA		58.5	49.4	54.2	73.01
	HbS% at the time of apheresis	NA		28.2	16.6	18.3	23.8
	Total Hemoglobin (g/L) prior hypertransfusion	120-170 (F)<br>130-180 (M)		94	96	103	90
	Total Hemoglobin (g/L) at the time of apheresis	120-170 (F)<br>130-180 (M)		100	102	90	92
	Reticulocyte (10^9/L) prior hypertransfusion	20-100		428	784	438	252.8
	Reticulocyte (10^9/L) at the time of apheresis	20-100		461	369.6	234	80.5
	Total bilirubin (μmol/L)* prior hypertransfusion	1.7-14.4		43	137	28	80
	Total bilirubin (μmol/L)* at the time of apheresis	1.7-14.4		29	78	82	48
	Lactate dehydrogenase (LDH) (U/L) prior hypertransfusion	130-250		648	532	283	574
	Lactate dehydrogenase (LDH) (U/L) at the time of apheresis	130-250		479	340	248	474
	Haptoglobine (g/dL) prior hypertransfusion	0.07-1.79		<0.08	<0.08	ND	ND
	Haptoglobine (g/dL) at the time of apheresis	0.07-1.79		<0.08	<0.08	ND	ND
	Ferritin (μg/L)** prior hypertransfusion	12-152		5144	3835	69	3137
	Ferritin (μg/L)** at the time of apheresis	12-152		ND	3189	ND	ND
	Aspartate amino transferase (ASAT)/Alanine amino transferase (ALAT) (U/L) prior hypertransfusion	9-40/7-50		52/17	28/41	52/44	41/13
	Aspartate amino transferase (ASAT)/Alanine amino transferase (ALAT) (U/L) at the time of apheresis	9-40/7-50		54/19	38/25	22/18	48/15
Hypertransfusion regimen	Weight (kg)			70	80.7	69	54.8
	RBC transfusion volume 3M prior harvest (mL)			6445	6248	2887	4347
	Number of ET 3M prior harvest			4	3	4	4
	RBC transfusion volume prior conditioning*** (mL)			3823	4173	2051	1665
	Number of ET prior conditioning			2	2	1	1
Transfusions	Number of ETs (RBC Transfusion Units (N Units per y))	Periods					
		pre GT	Y-3	13 (53)	8 (54)	10 (21)	12 (27)
			Y-2	11 (67)	10 (69)	12 (54)	11 (34)
			Y-1	19 (91)	15 (78)	19 (52)	14 (61)
		post GT	Y+1	1 (2)	1 (2)	13 (35)	4 (10)
			Y+2	0 (0)	0 (0)	3 (9)	0 (0)

NA, not applicable, ND, not determined

*P3 and P4 have homozygous Gilbert syndrome

**except for P3, the other patients are poorly compliant to iron-chelator treatment

***Conditioning started on average 2 months after the harvest

Supplementary Table 3. Blood parameters before and after gene therapy

		P1				P2				P3**				P4**			
	Normal Range	Baseline *	M3	M12	M24	Baseline *	M3	M12	M24	Baseline *	M3	M12	M24	Baseline *	M3	M12	M24
Red Blood Cell (10^{12}/L)	4.0-5.4 (F) 4.5-6.2 (M)	3.77	3.82	4.31	4.43	2.86	3.24	3.31	3.23	4.41	3.86	3.29	2.78	2.95	3.13	2.83	ND
Total Hemoglobin (g/L)	120-170 (F) 130-180 (M)	107.00	107.00	113.00	113.00	88	107.00	111.00	111.00	103.00	92.00	124.00	114.00	89.00	100.00	92.00	ND
Reticulocyte (10^9/L)	20-100	454.30	267.00	147.00	256.50	784,3	162.30	166.20	231.60	193.20	ND	64.10	125.10	252.80	284.80	ND	ND
White Blood Cell (10^9/L)	4-10	14.10	8.70	6.30	8.50	9.6	7.10	5.94	8.45	13.20	8.60	3.84	4.27	11.71	6.89	7.25	ND
Platelets (10^9/L)	150-450	159.00	107.00	193.00	188.00	422	222.00	384.00	285.00	440.00	370.00	114.00	199.00	363.00	248.00	304.00	ND
Ferritin (pmol/l) (µg/L)****	12-152	994.00	1781.00	2872.00	1623.00	3835	4309.00	3825.00	6694.00	17.00	43.00	159.00	160.00	2669.00	8093.00	5313.00	ND
Haptoglobin (g/L)	0.07-1.79	<0.08	10.37	1.46	ND	<0.08	<0.08	<0.08	<0.08	ND	<0.08	ND	<0.08	<0.08	<0.08	<0.08	ND
Lactate dehydrogenase (LDH) (U/L)	130-250	385.00	217.00	194.00	237.00	457.00	251.00	198.00	280.00	ND	275.00	426.00	414.00	429.00	354.00	323.00	ND
C reactive protein (mg/L)	<6.0	12.90	7.00	19.90	2.00	32.8	10.20	7.00	32.70	2.30	5.00	4.40	10.00	2.00	8.00	5.20	ND
Total bilirubin (µmol/L)***	1.7-14.4	43.00	21.00	16.00	22.00	148.00	41.00	51.00	100.00	28.00	28.00	19.20	25.00	97.00	48.00	53.00	ND
Conjugated bilirubin	0-5	7.00	5.00	ND	8.00	10.00	12.00	10.00	9.00	10.00	9.00	6.60	8.00	8.00	14.00	13.00	ND
Orthochromatic/ Polychromatic ratio in BM	1.9-2.3	1.2 (M2)	ND	ND	1.9	1.1 (M3)	ND	2.3	ND	ND	ND	ND	ND	1.2 (M4)	ND	1.4	ND

* under RBC exchange transfusion regimen at baseline
** still receiving RBC exchange transfusions
*** P3 and P4 have homozygous Gilbert syndrome
**** except for P3, the other patients are poorly compliant to iron-chelator treatment
M, months after GT; ND, not determined. Red: values higher than the normal range; blue: values lower than the normal range

Supplementary Fig. 1

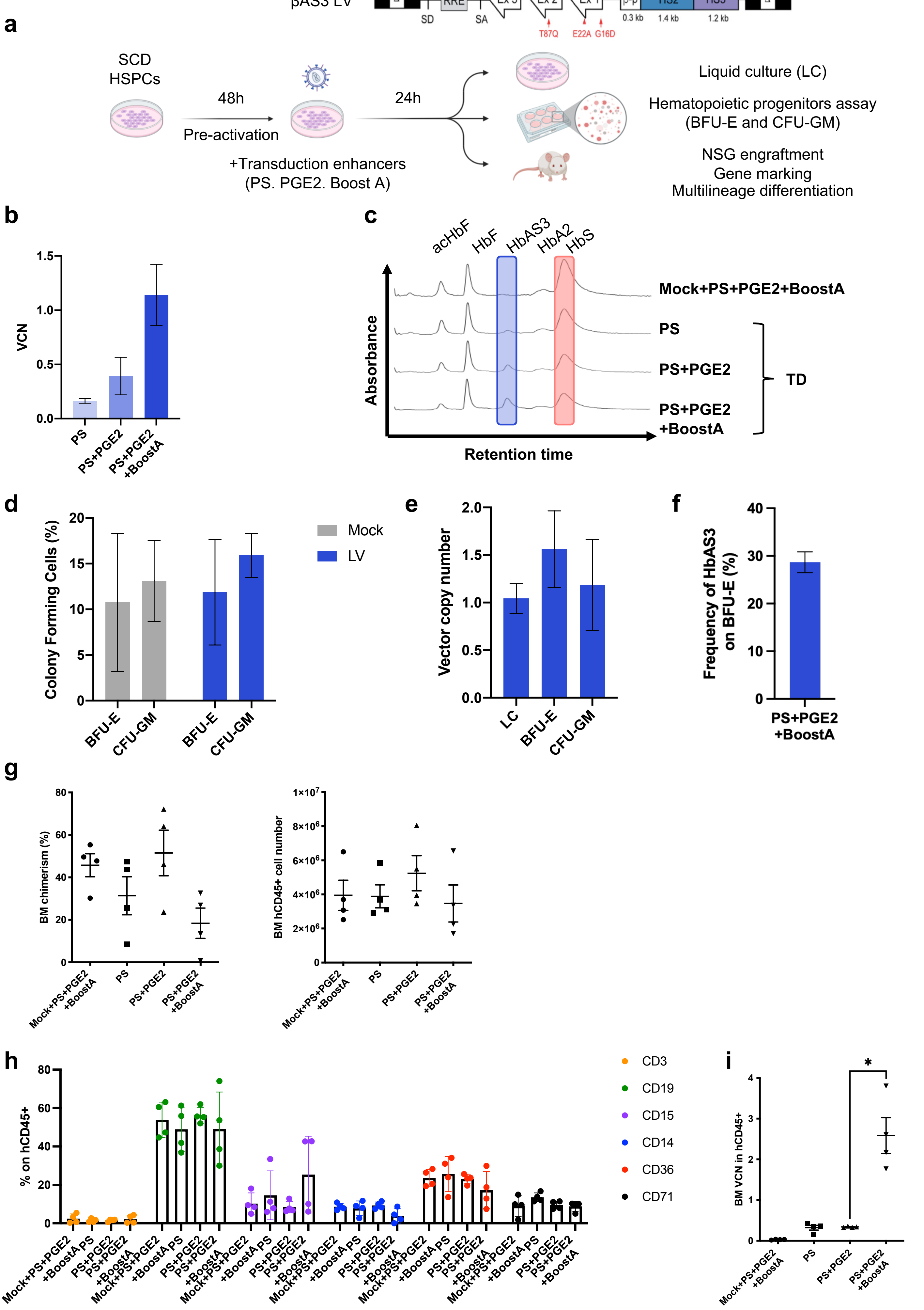

Supplementary Fig. 2

a

	P1	P2	P3	P4
**SAE** *Post-neutrophil engraftment up to last follow-up visit*				
Gastrointestinal disorders	-	-	-	1*
Febrile Neutropenia	1	-	-	-
Neoplasm (neuroendocrine tumor)	-	-	1	-
**AE** *Post-neutrophil engraftment up to last follow-up visit*				
Epistaxis	-	-	-	1
Blood and lymphatic system disorders	1	1	2	
Skin disorders	2	1	-	-
Liver disorder	-	-	2	-
Infection°	1	1	4	2

* Mucositis
° Microbiologically or radiologically documented

b

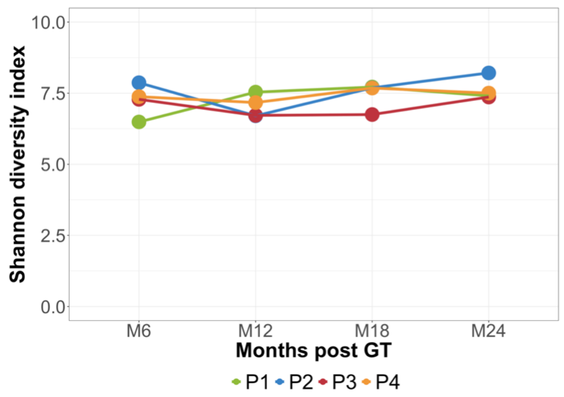

d

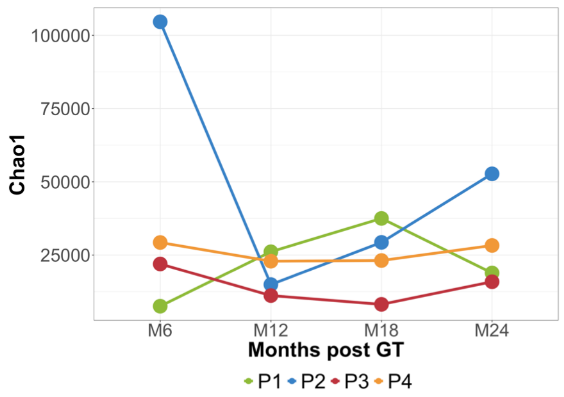

c

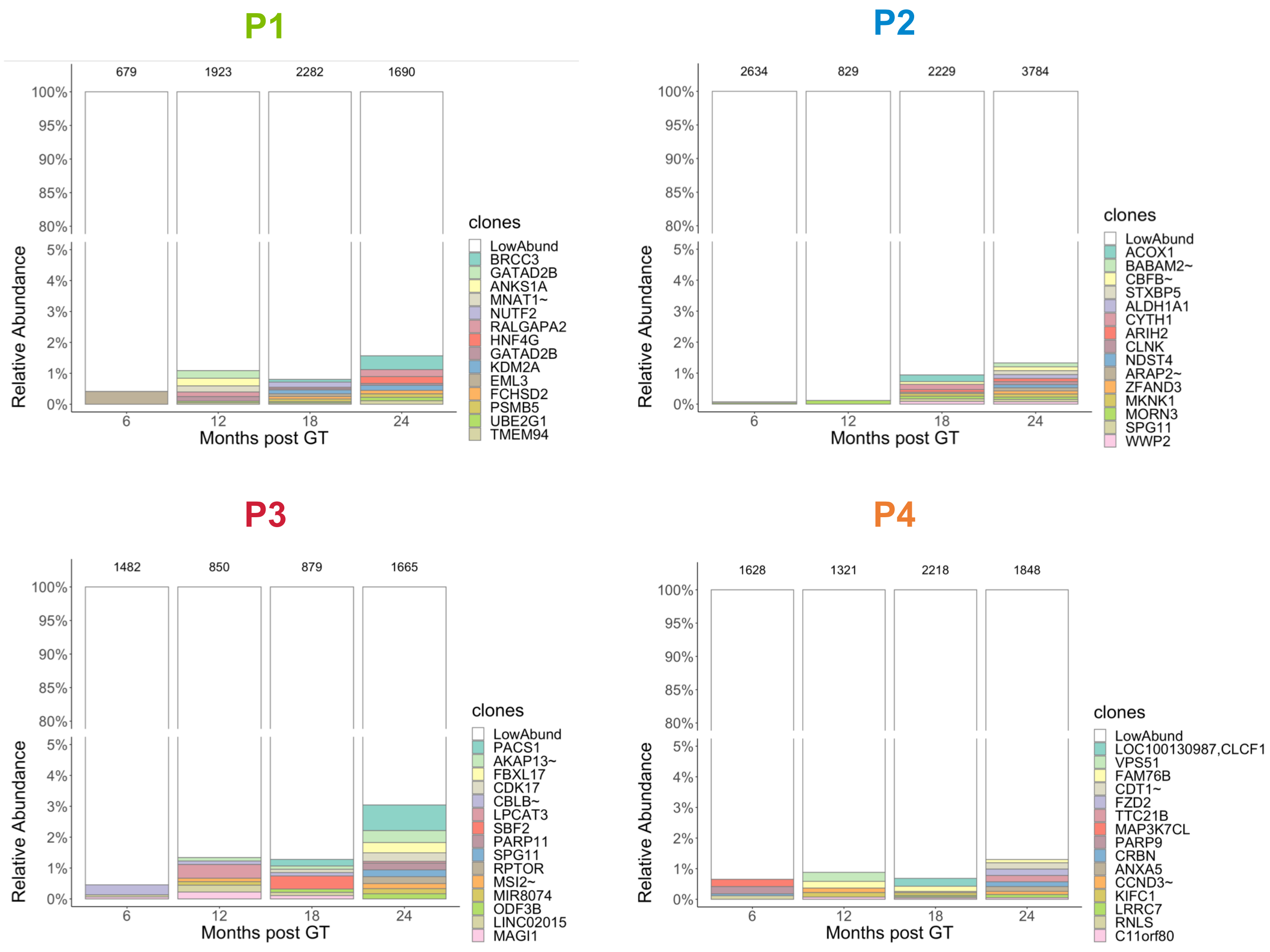

Supplementary Fig. 3

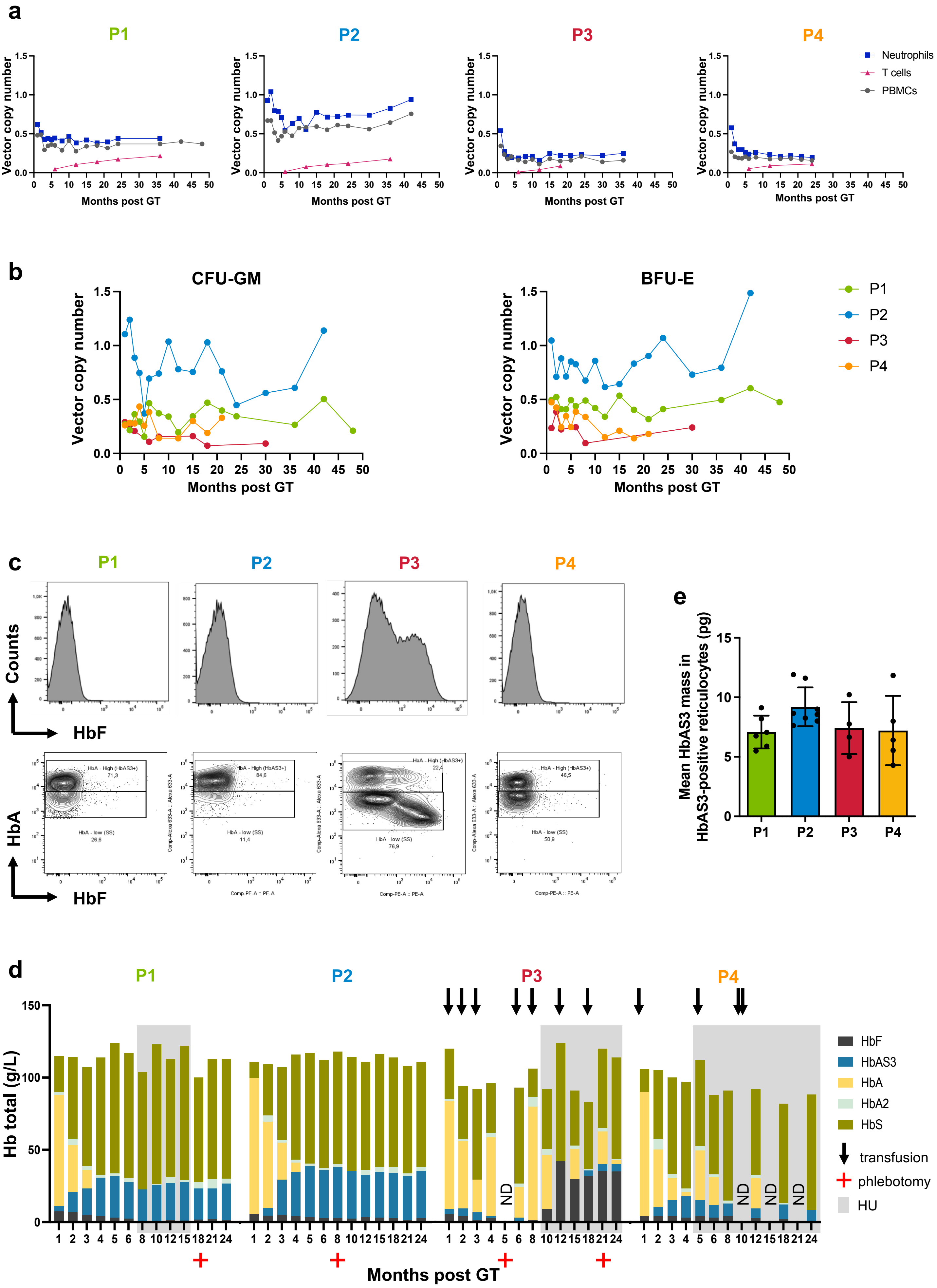

Supplementary Fig. 4

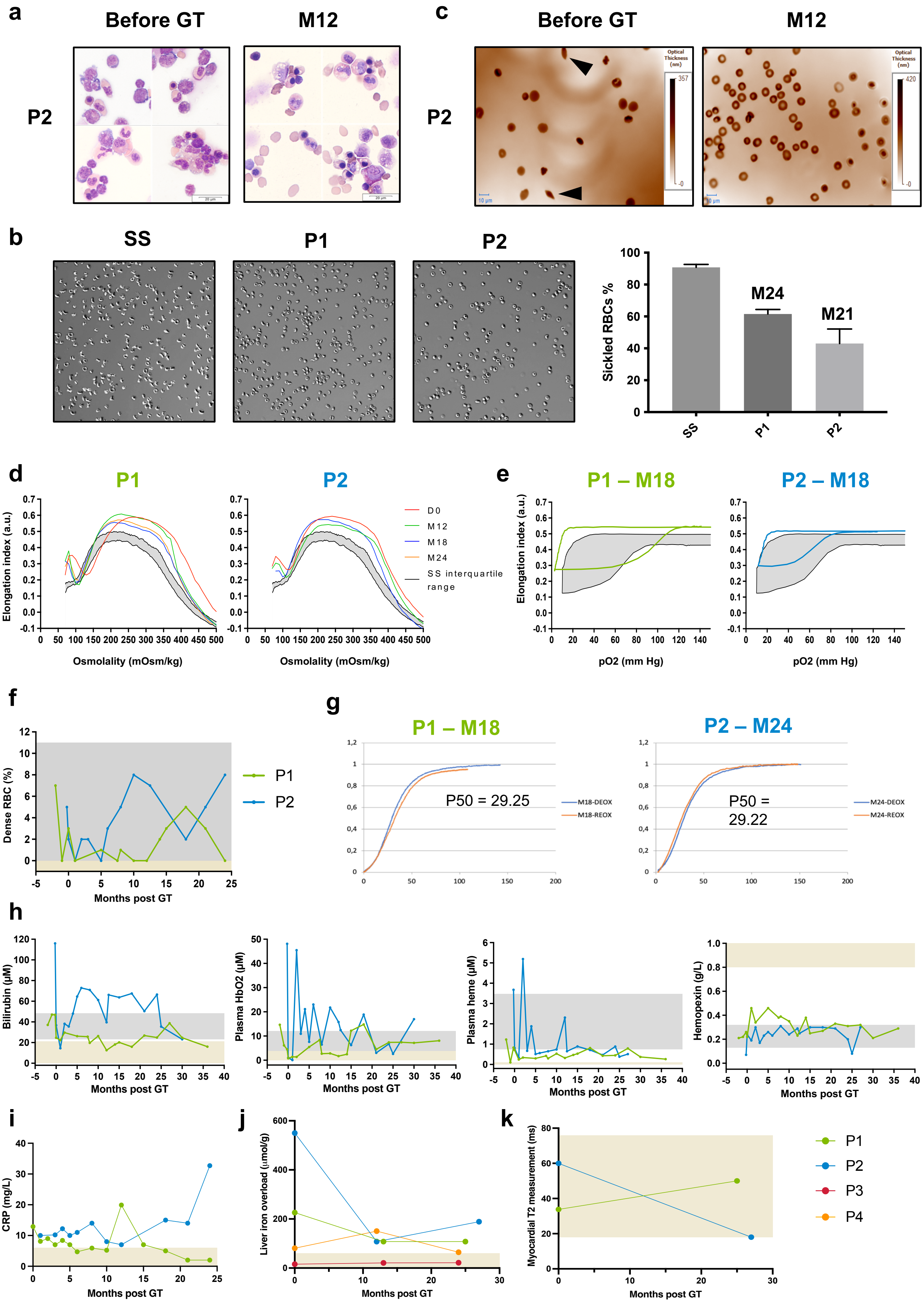

Supplementary Fig. 5

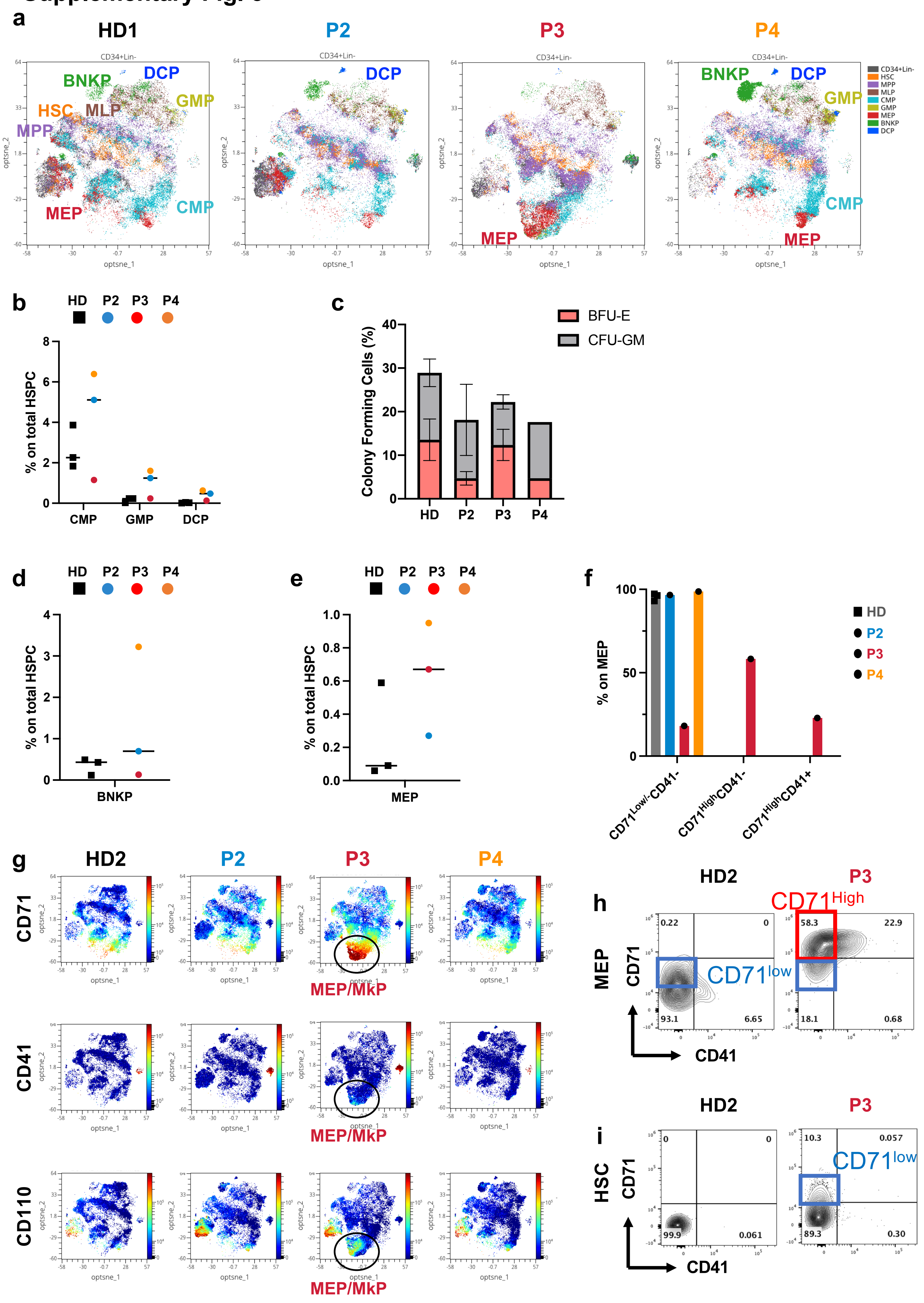

Supplementary Fig. 6

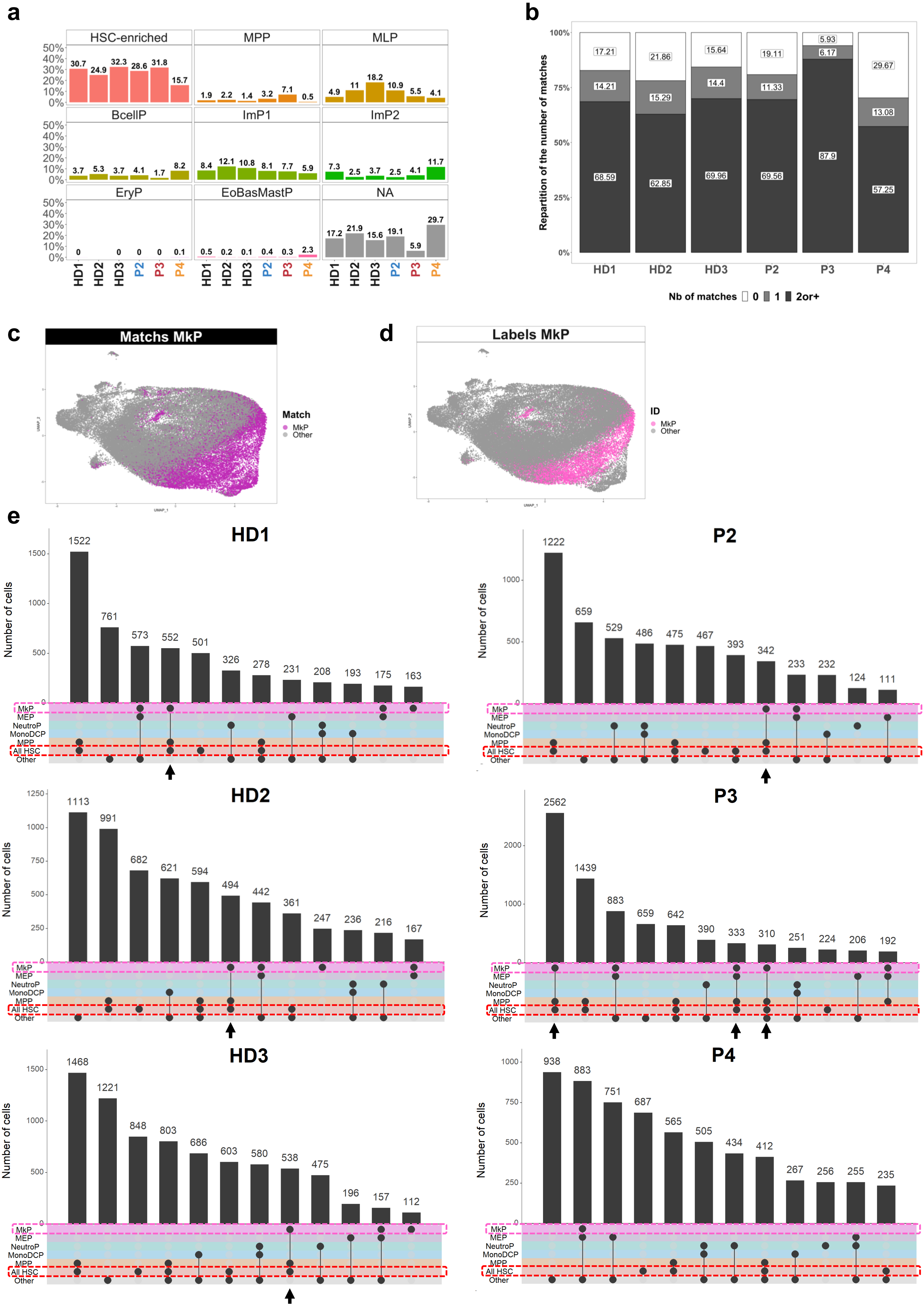

Supplementary Fig. 6

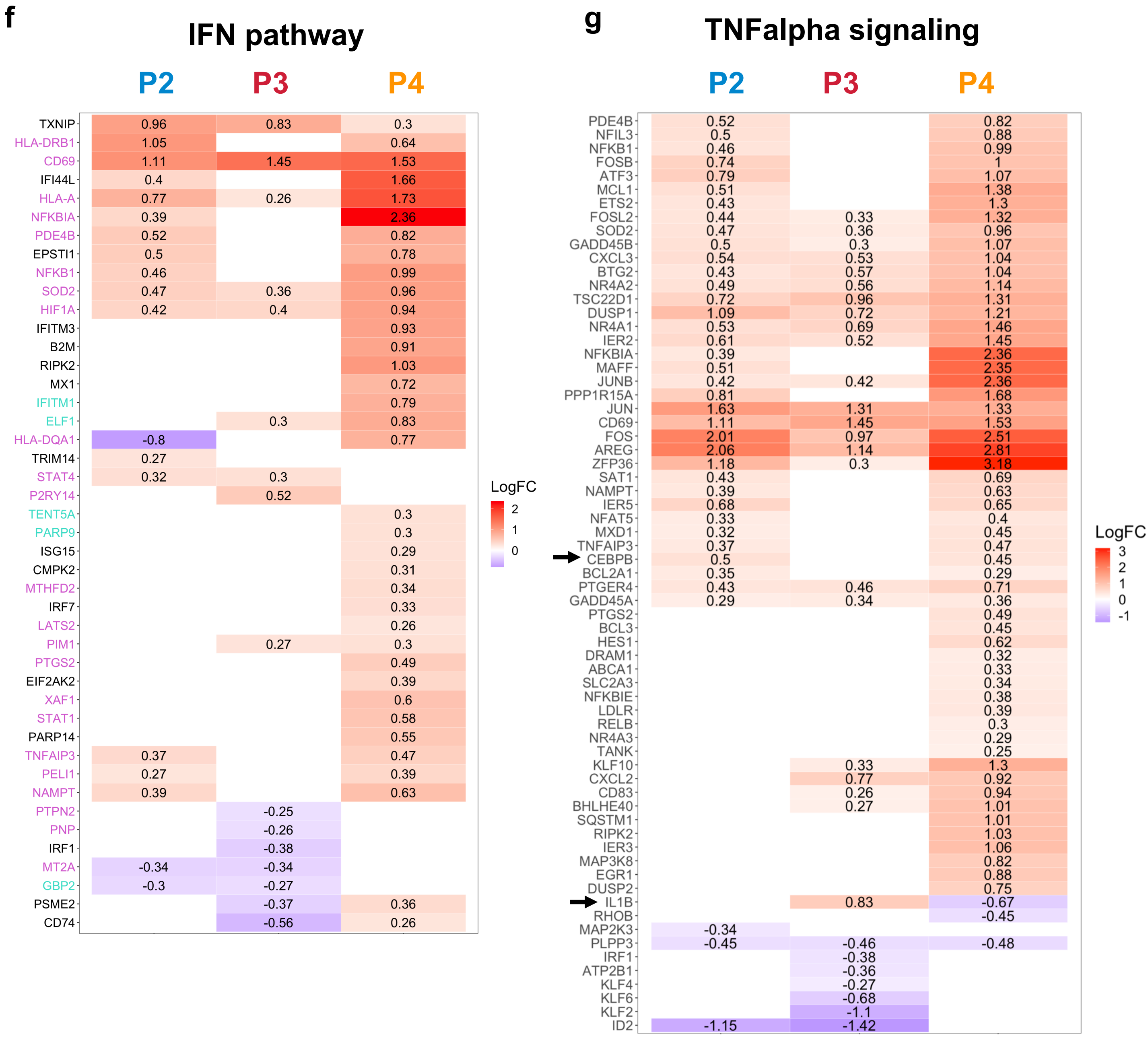

Supplementary Fig. 7

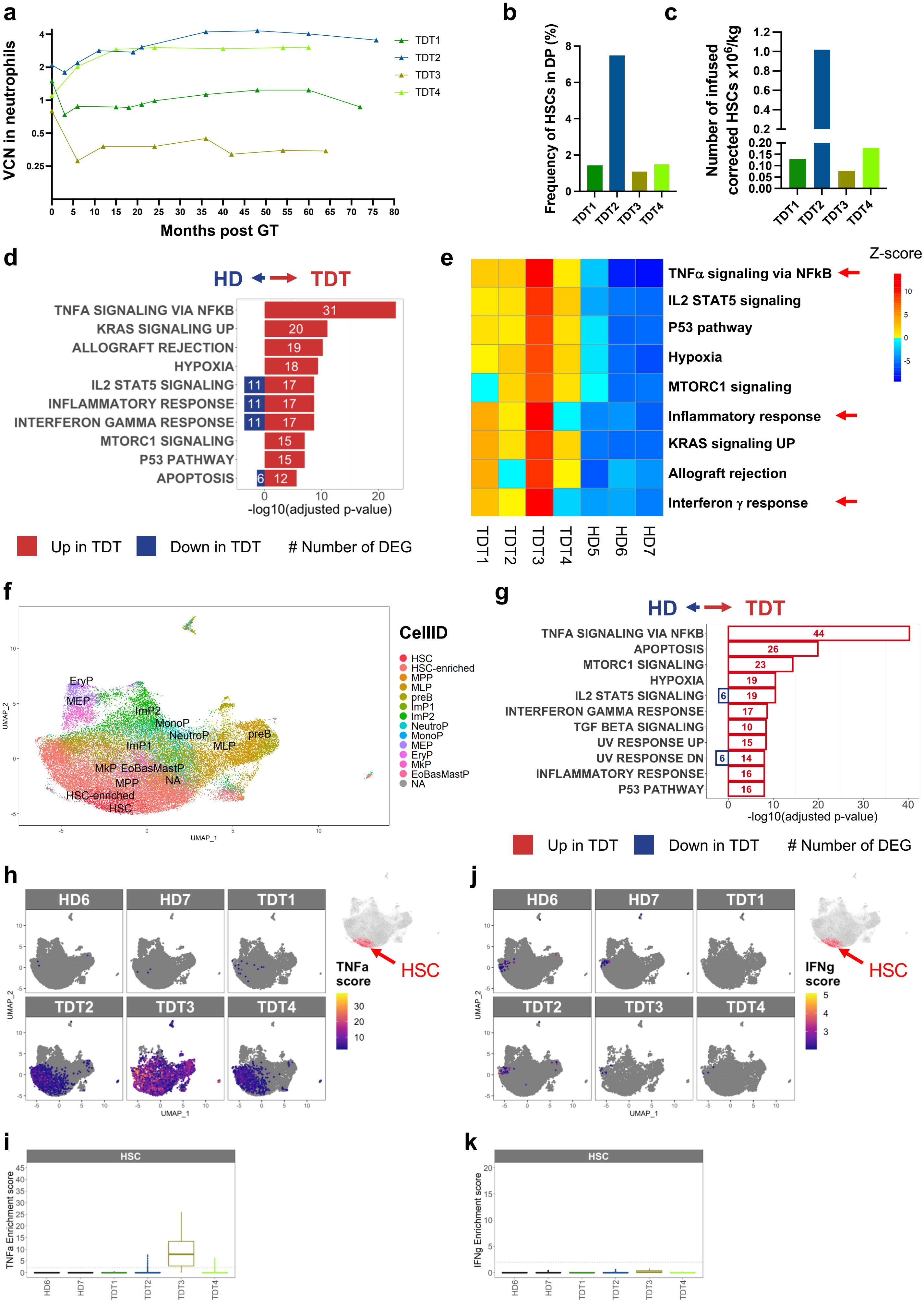

Supplementary Fig. 8

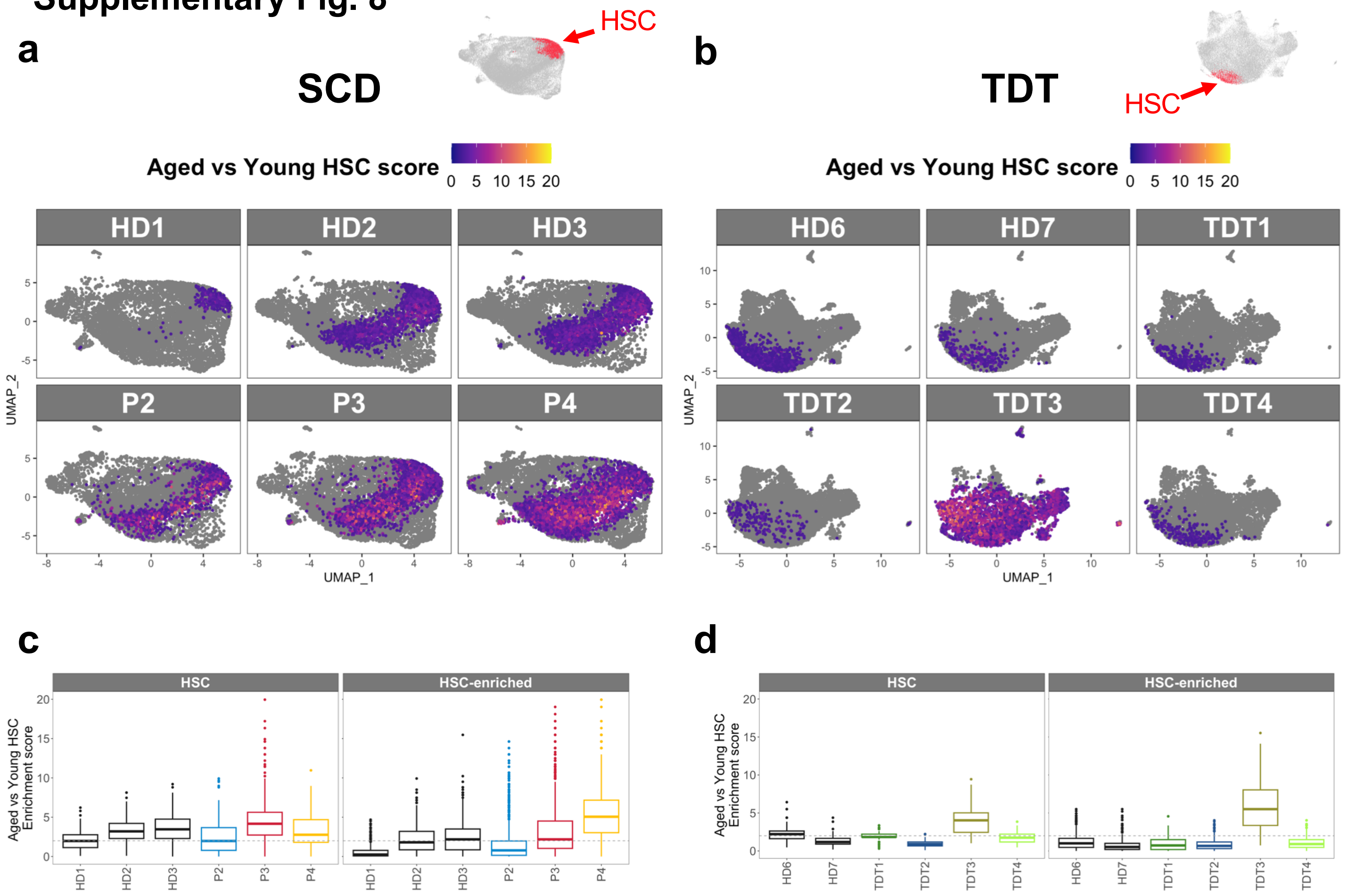

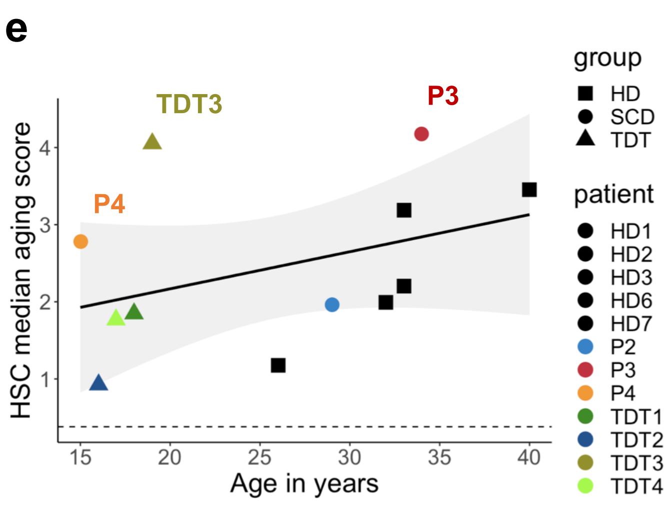

	HD	HD + Patients
**r**	0.89	0.389
**p**	0.045	NS

DREPAGLOBE protocol versions

VERSION	Inclusion criteria modification	Other modification
V1.1 11 October 2018		
V2.1 30 April 2019	No inclusion criteria modification	New clinical departments involvement
V3.0 27 July 2019	No inclusion criteria modification	Reduction period pre GT
V4.0 23 October 2019	Inclusion criteria modification	
V5.0 10 April 2020		Modification of the visit schedule
V6.0 9 April 2021	No inclusion criteria modification	
V7.0 17 August 2021		New clinical departments involvement
V8.0 5 January 2022		Modification of the visit schedule<br>New clinical departments involvement

« A Phase 1/2 Open Label Study Evaluating the Safety and Efficacy of Gene Therapy of the Sickle Cell disease by Transplantation of an Autologous CD34+ enriched cell fraction that contains CD34+ cells transduced ex vivo with the GLOBE1 lentiviral vector expressing the βAS3 globin gene (GLOBE1 βAS3 Modified Autologous CD34+ Cells) in Patients with Sickle Cell Disease (SCD) »

DREPAGLOBE

INTERVENTIONAL RESEARCH PROTOCOL

RELATING AN INNOVATING EXPERIMENTAL THERAPY DRUG FOR HUMAN USE

Version N° **8.0 of 05/01/2022**

Project code number: P171006J EUDRACT No.: 2018-001968-33

**Coordinating Investigator:**<br>**Pr Marina CAVAZZANA**<br>Department of Biotherapy<br>Necker-Enfants Malades Hospital,<br>149 rue de Sèvres, 75743 Paris, Cedex 15,<br>Email: m.cavazzana@aphp.fr	
**Scientific Directors:**<br>**Dr Elisa MAGRIN**<br>Department of Biotherapy<br>Necker-Enfants Malades Hospital,<br>149 rue de Sèvres<br>75743 Paris, Cedex 15<br>Email:elisa.magrin@aphp.fr	**Pr Pablo BARTOLUCCI**<br>Hôpital Henri-Mondor<br>Service de Médecine interne<br>51 av mal de Lattre de Tassigny.<br>94010 Créteil, France
**Sponsor :**<br>Assistance Publique-Hôpitaux de Paris : Clinical Research and Development Department<br>**Project manager : Ophélie ROGIER**<br>Mail : ophelie.rogier@aphp.fr<br>Hôpital Saint Louis<br>1 avenue Claude Vellefaux, 75010 PARIS	
**Entity responsible for monitoring the trial**:<br>Unité de Recherche Clinique (URC) PARIS CENTRE<br>Hôpital Necker<br>**Project manager :Jinmi BAEK**<br>Mail: jinmi.baek@aphp.fr	

Clinical Research and Innovation Delegation (DRCI) Hôpital Saint Louis 75010 PARIS

1 SUMMARY

Full title	A Phase 1/2 Open Label Study Evaluating the Safety and Efficacy of Gene Therapy of the Sickle Cell Anemia by Transplantation of an Autologous CD34+ enriched cell fraction that contains CD34+ cells transduced ex vivo with the GLOBE1 lentiviral vector expressing the βAS3 globin gene (GLOBE1 βAS3 Modified Autologous CD34+ Cells) in Patients with Sickle Cell Disease (SCD)
Acronym	DREPAGLOBE
Coordinating Investigator	Professor Marina CAVAZZANA<br>Biotherapy Department, Necker-Enfants Malades Hospital; 149, Rue de Sèvre, 75015 Paris, France
Sponsor	Assistance Publique-Hôpitaux de Paris
Scientific justification	Improvements in the care of patients with SCD have been few and gradual over past decades (Piel 2017, N Engl J Med). The only current treatment that is potentially curative (restoration of normal hematopoiesis) for SCD is HSCT but it is applicable to a small minority of patients globally due to its high cost, toxicities including procedure-related mortality, and limited availability of suitable donors (Bernaudin, 2012, Blood).<br>The DREPAGLOBE drug product is an autologous stem cell gene therapy for SCD that is designed to permanently cure this debilitating illness without the immunological complications or need for immune suppression of current allogeneic HSCT approaches. The insertion of anti-sickling globin genes is anticipated to decrease polymerization of sickle fibrils and the subsequent sickling of RBC to decrease or prevent the significant morbidity and increased mortality experienced by patients with SCD.
Main objective and primary endpoint	The primary objective of the study is to assess the initial safety and tolerability of treatment with DREPAGLOBE drug product, including the mobilization procedure with Plerixafor, conditioning regimen and transplantation with GLOBE1 βAS3 lentiviral vector gene modified autologous CD34+cells in up to 10 severe SCD patients.

This document is the property of DRCI / AP-HP. All reproduction is strictly prohibited.
Version date: April 2018

	<u>The primary endpoints</u> of this study are safety.<br>Safety in the first 6 months following IV infusion of DREPAGLOBE drug product, as assessed by:<br>• Incidence of transplant related mortality up to 100 days post treatment<br>• Incidence of the need for rescue autologous bone marrow transplant up to 100 days post treatment<br>• Frequency and severity of clinical AEs and laboratory parameters<br>• Incidence of RCL<br>Incidence of clinically detectable malignancy and/or abnormal clonal dominance assessed as related to study treatment
Secondary objectives and endpoints	The secondary objectives of the study are:<br>• To assess the initial efficacy of treatment with GLOBE1 βAS3 lentiviral vector modified autologous CD34+ stem cells.<br>• To assess the long-term safety and efficacy of treatment with the DREPAGLOBE drug product<br><br>The secondary efficacy endpoints, as assessed in the first 6 months following transplantation, are:<br>• In vivo engraftment through hematopoietic reconstitution after IV infusion of the DREPAGLOBE drug product, i.e. neutrophil and platelet recovery (neutrophil recovery defined as the first of three consecutive days with an ANC of > 500/µL and platelet recovery defined as the first of three consecutive days with a platelet count of > 20000/µL sustained without platelet transfusion for at least seven days)<br>• Percentage HbAS3.<br>The other endpoints are long-term safety and efficacy (throughout the 24-month follow-up phase) as measured by:<br>• Monitoring the frequency and severity of adverse events based on NCI CTCAE v4.03<br>• Absence of RCL

This document is the property of DRCI / AP-HP. All reproduction is strictly prohibited.
Version date: April 2018

	• Absence of clinically detectable malignancy or abnormal clonal dominance assessed as related to study treatment<br>• Protein expression through percentage of anti-sickling Hb<br>• Percentage HbAS3
Design of the trial	The study is a prospective, baseline-controlled, non-randomized, open-label, Phase 1/2 clinical study to assess the safety, and efficacy of the gene therapy by Transplantation of an Autologous CD34+ enriched cell fraction that contains CD34+ cells transduced ex vivo with the GLOBE1 lentiviral vector expressing the βAS3 globin gene.
Population of trial subjects	The population enrolled in this study is constituted by 2 cohorts: cohort 1 including 3 adult patients (aged 18 to 35 years), then cohort 2 will include up to 7 additional patients aged 12 to 20 years. They are all diagnosed of HbSS or S-beta zero thalassemia with severe, life-threatening SCD who are eligible for an allogeneic hematopoietic stem cell trans plant (HSCT) ; but do not have a suitable, willing, 10/10 matched human leukocyte antigen (HLA)-identical sibling donor.
Inclusion criteria	Individuals eligible to participate in this study must meet all of the following criteria:<br>• Age 12-20 years<br>• Diagnosis of HbSS or S-beta zero thalassemia by Hb electrophoresis or genetic analysis<br>• Clinical history or ongoing evidence of severe sickle cell anemia with one **OR** more of the following clinical complications demonstrating disease severity:<br>1. At least 3 vaso occlusive crises requiring hospitalization, under hydroxyurea or transfusion, within 2 years prior to enrollment<br>2. One severe acute chest syndrome (ACS) hospitalized in intensive care unit<br>3. At least 2 episodes of ACS within the prior 3 years), including one under HU.

This document is the property of DRCI / AP-HP. All reproduction is strictly prohibited.
Version date: April 2018

	4. Acute priapism (at least 2 episodes > 3h in the preceding year or in the year prior to the start of a regular transfusion program), OR stuttering priapism ≥ 1 by week under sickle cell treatment (HU, transfusion or phlebotomy).<br>5. Cerebral vasculopathy confirmed by MRA (magnetic resonance angiography) without Moya-moya<br>6. Presence of sickle cell cardiomyopathy documented by Doppler echocardiography (left ventricular ejection fraction (LVEF) <55% AND tricuspid regurgitation velocity >2.5m/s on cardiac echocardiograph),<br>7. Tricuspid regurgitation velocity >2.8m/s on cardiac echocardiograph without pulmonary hypertension confirmed by right heart catheterization (mPAP<25mmHg)<br><br>• Failed hydroxyurea (HU) therapy, were unable to tolerate HU therapy, or, if 18 years of age or older, have actively made the choice to not take the recommended daily HU regimen. Inadequate clinical response to HU, defined as any one of the following outcomes, while on HU for at least 3 months: 2 or more acute sickle pain crises requiring hospitalization, no rise in Hb >1.5 g/dl from pre-HU baseline or requires transfusion to maintain Hb > 6.0 g/dL, Has an episode of ACS despite adequate supportive care measures.<br>• Karnovsky/Lansky performance score ≥ 60 %<br>• Sexually active patients must be willing to use an acceptable method of double-barrier contraception for at least 12 months post-infusion (beyond 12 months at the discretion of the investigator)<br>procedure for obtaining consent (adults, dependent minors, to give their consent, affiliation of a social security regime (or exemption).

This document is the property of DRCI / AP-HP. All reproduction is strictly prohibited.
Version date: April 2018

Exclusion criteria	Individuals who meet any of the following exclusion criteria will not be eligible to participate in the study:<br>• Chromosomal (karyotyping) or molecular anomalies (detected by NGS) (i.e. 7 chromosomal monosomy)<br>• Existence of a matched sibling donor<br>• Patients who have started new treatment for SCD within 6months of enrollment<br>• Hematologic evaluation: Leukopenia (WBC < 3000 /µL) or neutropenia (ANC < 1000 /µL) or thrombocytopenia (platelet count < 100,000 /µL) (not due to an erythrapheresis procedure)<br>• PT/INR or PTT > 1.5 times upper limit of normal (ULN) or clinically significant bleeding disorder<br>• Evaluations within 6 months prior to screening visit:<br>1. ALT or AST > 3 times ULN<br>2. Liver Cirrhosis suspicion on echography, CT scan or MRI AND confirmed by histology<br>3. Cardiac evaluation: LVEF < 40% by cardiac echocardiogram or by MUGA scan or clinically significant ECG abnormalities<br>4. Stroke with significant CNS sequelae i.e., Rankin > 2<br>5. Lung interstitial infiltrate AND Forced Vital Capacity less than 70% AND DLCO less than 60% at steady state<br>6. Confirmed pulmonary hypertension defined by a right heart catheterization (PAPm>25mmHg). Right heart catheterization is required if tricuspid regurgitation velocity >2.8m/s on cardiac echocardiograph OR >2.5m/s with an abnormal Brain Natriuretic Peptide dosage or an important decrease in transcutaneous Hb O2 saturation during the 6 minutes' walk test.<br>• Seropositivity for HIV (Human Immunodeficiency Virus), HCV (Hepatitis C Virus), HTLV-1 (Human T-Lymphotropic Virus), or active Hepatitis B Virus, or active infection by CMV or parvovirus B19, based on positive blood PCR.<br>• Pregnancy or breastfeeding in a postpartum female

This document is the property of DRCI / AP-HP. All reproduction is strictly prohibited.
Version date: April 2018

	• Any current cancer or prior history of a malignant disease, with the exception of curatively treated non-melanoma skin cancer<br>• Immediate family member with an established or suspected Familial Cancer Syndrome<br>• Diagnosis of significant psychiatric disorder of the subject that could seriously impeded the ability to participate in the study<br>• Patients who failed previous HSCT and are severely ill<br>• Any clinically significant active infection<br>• Participation in another clinical study with an investigational drug within 30 days of screening<br>• Any condition, based on perspective of the medical monitor and treating investigator, which may lead to increased safety risk or inability to comply with the protocol
Investigational medicinal product(s)	Each patient will receive a single intravenous infusion of DREPAGLOBE drug product at dose range of 3 to20 million CD34+ cells/kg post-transduction.<br>Source of CD34+ will be :<br>1) CD34 + mobilized with Plerixafor after a single or multiple harvests<br>2) Bone- Marrow<br>• The DREPAGLOBE drug product consists in autologous CD34+ hematopoietic stem cells transduced with Self-inactivating lentiviral vector (GLOBE1 βAS3) encoding the human βAS3-globin gene and suspended in HSA (5% Albunorm™) in the final immediate container for the intended medical use. All subjects are to receive the DREPAGLOBE drug product on Day 1 via IV infusion according to applicable SOPs, with vital signs being monitored concurrently. The minimum dose to be administered is 3.0×10^{6} CD34+ cells/kg.
Interventions added for the trial	• Exchange transfusion before the mobilization procedure<br>• Mobilization by Plerixafor

This document is the property of DRCI / AP-HP. All reproduction is strictly prohibited.
Version date: April 2018

	• Apheresis/ conditioning regimen<br>Infusion with GLOBE1 βAS3 lentiviral vector
Risks added by the trial	D
Scope of the trial	This study consists of 5 phases:<br>• Screening<br>• Mobilization<br>• Myeloablation<br>• Treatment (up to 1 week)<br>• Follow-up (up to 24 months)<br>Up to 10 evaluable patients with severe SCD will be treated in the study to receive a single IV dose of the DREPAGLOBE drug product.<br>There will be a sequential enrolment.<br><br>The first three patients enrolled will be adults (*The clinical trial will be introduced in priority of patients previously enrolled in the Drepamob protocol/ Eudract N° 2014-001650-42).* The second patient will be enrolled when the previous patient has safely reconstituted the peripheral hematological compartment (i.e., ANC > 500 /µL), estimated to occur approximately 4-6 weeks post-treatment. The same process will be performed for the third patient.<br>If we assess that the first three treated patients present good clinical results and benefits (Stable hemoglobin production over time), SCD patients between 12 and 20 years old will be enrolled sequentially (no specific cohort for pediatric and adults patients).<br>Treatment will not be initiated in pediatric patients until the first three adult patients have been treated after ANC recovery.
Number of subjects included	Up to 10 evaluable patients will be treated under this protocol.
Number of sites	6 sites:<br>- N°001: Department of Biotherapy, Necker-Enfants Malades Hospital (Pr CAVAZZANA)

This document is the property of DRCI / AP-HP. All reproduction is strictly prohibited.
Version date: April 2018

	- N°002: Department of Adult Hematology, Necker-Enfants Malades Hospital (Pr HERMINE)<br>- N°003: Adult Intensive care Unit, Necker-Enfants Malades Hospital (Dr LAMHAUT)<br>- N°004: CIC, Necker-Enfants Malades Hospital (Dr SEMERARO)<br>- N°005: Paediatric Immunohematology and Rheumatology Unit, (UIHR), Necker-Enfants Malades Hospital (Dr CASTELLE)<br>- N°006: Department of internal medicine, Henri-Mondor Hospital (Pr BARTOLUCCI).<br><br>First of all, there will be one site for the manufacture of the Drepaglobe drug product: (P.I.: Marina CAVAZZANA) Department of Biotherapy, Necker-Enfants Malades Hospital; 149, Rue de Sèvres, 75015 Paris, France (n°001).<br><br>**For adult patients:**<br>The mobilization will be at the department of Adult Hematology, Necker Enfant-Malades Hospital (n°002) or Adult Intensive care Unit, Necker Enfant-Malades Hospital (n°003).<br>The conditioning and the gene therapy treatment will be at the department of Adult Hematology, Necker Enfant-Malades Hospital (n°002)<br><br>According to patient's clinical condition, screening visit, baseline visit, exchange transfusion phase and follow-up visits will be realized in in one of the sites listed above (n°001, 002, 003, 004, 006), except at the UIHR (n°005).<br><br>**For minor patients:**<br>The mobilization, the conditioning and the gene therapy treatment will be at the UIHR (n°005).<br>According to patient's clinical condition, screening visit, baseline visit, exchange transfusion phase and follow-up visits will be realized at the UIHR (n°005), the Department of

This document is the property of DRCI / AP-HP. All reproduction is strictly prohibited.
Version date: April 2018

	Biotherapy (n°001) or at the CIC (n°004) Necker-Enfants Malades Hospital.
Duration of the trial	Inclusion period : 33 months<br>participation period post transplantation : 24 months<br>Study total duration : 66 months
Statistical analysis	No formal statistical tests will be performed because of the small sample size.<br>Tabulation will be produced for appropriate demographic, baseline, efficacy and safety parameters. Categorical data will be summarized using frequency and percent, while continuous data will be summarized using descriptive statistics (i.e., number of observations, mean, median, standard deviation [SD], minimum, and maximum). By-subject listings of data for all completed and discontinued subjects will be provided.<br>For disease-specific biological parameters and clinical events, including number of VOC events, acute chest syndrome events, strokes or transient ischemic attacks and RBC transfusion, baseline will be defined as the average of these parameters over the 3 years prior to study entry. For other change from baseline analyses, baseline will be defined as the value closest to, but prior to transplant.
Sources of funding for the trial	BioMarin Pharmaceutical Inc.
Trial will have a Data Monitoring Committee	*Yes*

This document is the property of DRCI / AP-HP. All reproduction is strictly prohibited.
Version date: April 2018
