# Supplementary material for: Severe inflammation and lineage skewing are associated with poor engraftment of engineered hematopoietic stem cells in patients with sickle cell disease
Source: Nat Commun. 2025 Apr 1;16:3137. doi: 10.1038/s41467-025-58321-4 (PMC11961595; doi:10.1038/s41467-025-58321-4)
Supplement: Supplementary file 2 — Description of Additional Supplementary Files [file 41467_2025_58321_MOESM2_ESM.pdf]

Description of Additional Supplementary Files

Supplementary Data 1: Gating strategy to assess HSCs. Linear FSC-A versus linear SSC-A was used to gate the human HSPC. Within the human cells, FSC-A versus FSC-H was used to gate on single cells. In the single cell population, SSC-A versus Fixable Viability Stain (FVS) 575V was used to gate on the FVS 575V negative (live) cells. Within the live cells, CD34 APC-Cy7 versus Lineage PE was used to gate on CD34+Lin-, then the CD133 PE-Dazzle-594 vs CD38 BV605 to gate on CD133+CD38- and the CD90 PE-Cy5 vs CD45RA BV711 to gate on CD90+CD45RA- to determine the HSC subpopulation.

Supplementary Data 2: Gating strategy to assess HbAS3 expression in single RBCs and mean amount of HbAS3 per CD71+ reticulocyte. Doublet exclusion was performed on SSC-A vs. FSC-A dot plot by selecting individual RBCs and then on SSC-H vs. FSC-H followed by SSC-W vs. SSC-H to select single cells. The selection of patients' own RBCs (in case of transfusion) was done on FSC-A vs. HbS-PB-A to select HbS-positive cells. A control of the day, made from a mix of RBCs from an untransfused SS individual and RBCs from a AS individual was used to set the gates "HbA-low" and HbA-high" on a HbS-PB-A vs. HbA-AF647-A dot plot, based in the AF-647 (anti-HbA antibody) fluorescence intensity. The gate "HbA-low" corresponds to SS RBCs for which the AF-647 fluorescence only comes from the intracellular HbA2. The gate "HbA-high" corresponds to AS RBCs, for which the AF-647 fluorescence comes from both HbA2 and HbA. The same gates with the same settings were then applied to HbS-positive RBCs in GT patients acquired the same day. In P1, P2, P3 and P4, the "HbA-low" gate, which exhibits similar AF-647 mean fluorescence intensity as SS-RBCs, corresponds to the RBC% without detectable intracellular HbAS3. In these patients, the gate "HbA-high", which exhibits higher AF-647 MFI than SS-RBC, corresponds to RBC containing intracellular HbA2 and detectable HbAS3, giving the percentage of HbAS3-positive RBCs. From the HbS-positive cells (gated on FSC-A vs. HbS-PB-A) reticulocytes were selected on FSC-A vs. CD71-PE-A and the similar gating strategy as above was applied to assess the percentage of HbAS3-positive reticulocytes

Supplementary Data 3: Gating strategy to assess orthochromatic and polychromatic erythroblasts. Linear FSC-A versus linear SSC-A was used to gate the mononuclear cells. Within the human cells, FSC-W versus FSC-A was used to gate on single cells. In the single cell population, FSC-A versus 7-AAD was used to gate on the 7-AAD negative (live) cells. Within the live cells, GPA-Pacific-Blue was used to gate on GPA+ and the CD49d and Band3 to determine the orthochromatic and polychromatic erythroblasts.

Supplementary Data 4: Table of ten cells (randomly taken in P3 dataset) showing the -log10(p-value) calculated for each HSPC reference signature. For each cell, Cell ID is defined with the top -log10(p-value) (bold framed). For each cell, orange color indicates the various cell type signatures that significantly match (-log10(p-value)>1.3) and the number of Cell Match is indicated at the bottom of the table.

Supplementary Data 5. Gene signature of the mixed All HSC-MkP population.

Supplementary Data 6. DEGs between HSCs from each patient and HD HSCs (log fold-change)
