# Supplementary material for: Severe inflammation and lineage skewing are associated with poor engraftment of engineered hematopoietic stem cells in patients with sickle cell disease
Source: Nat Commun. 2025 Apr 1;16:3137. doi: 10.1038/s41467-025-58321-4 (PMC11961595; doi:10.1038/s41467-025-58321-4)
Supplement: Supplementary file 3 — Supplementary Datasets 1-4 [file 41467_2025_58321_MOESM3_ESM.pdf]

Supplementary Data 1

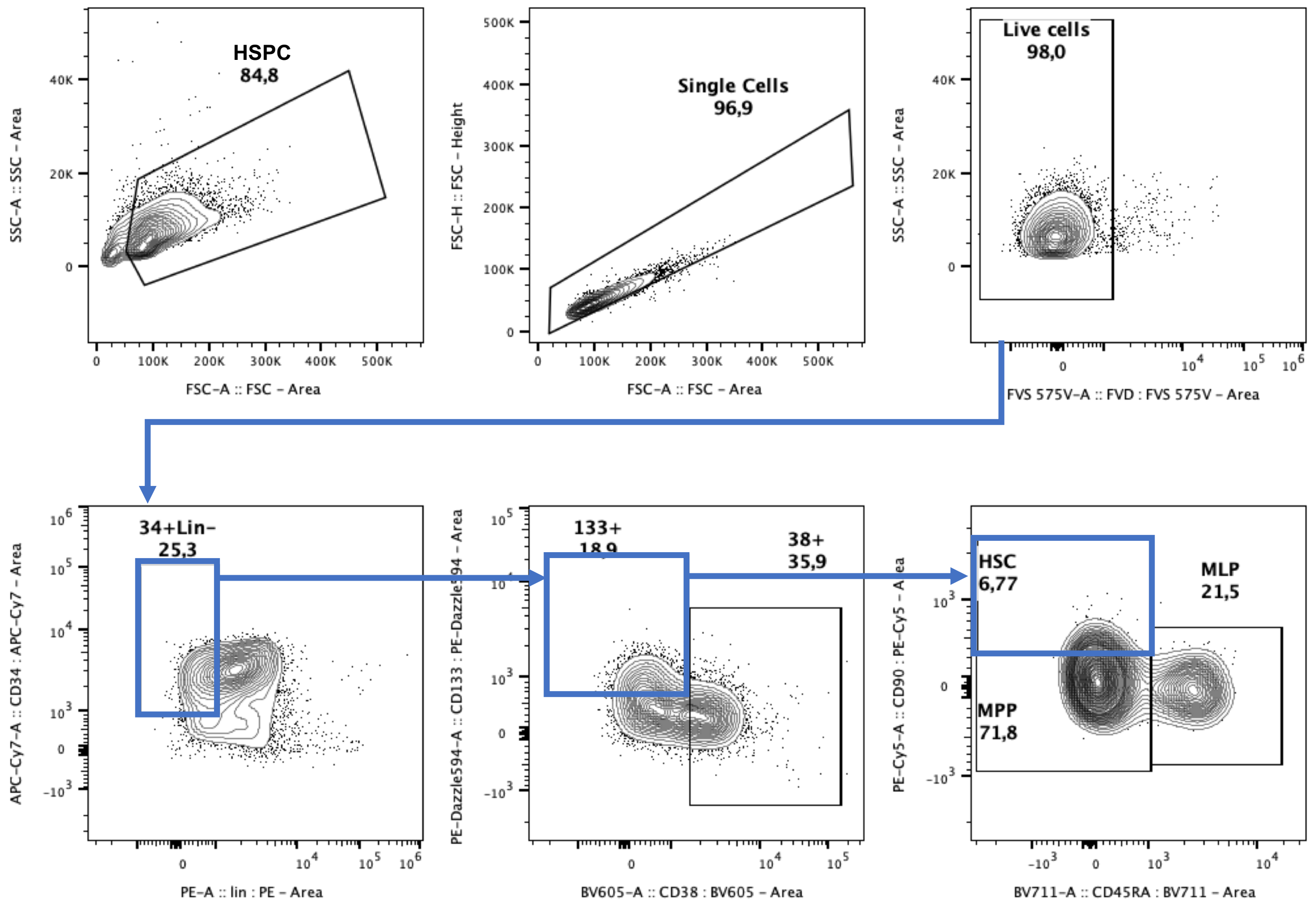

Supplementary Data 2

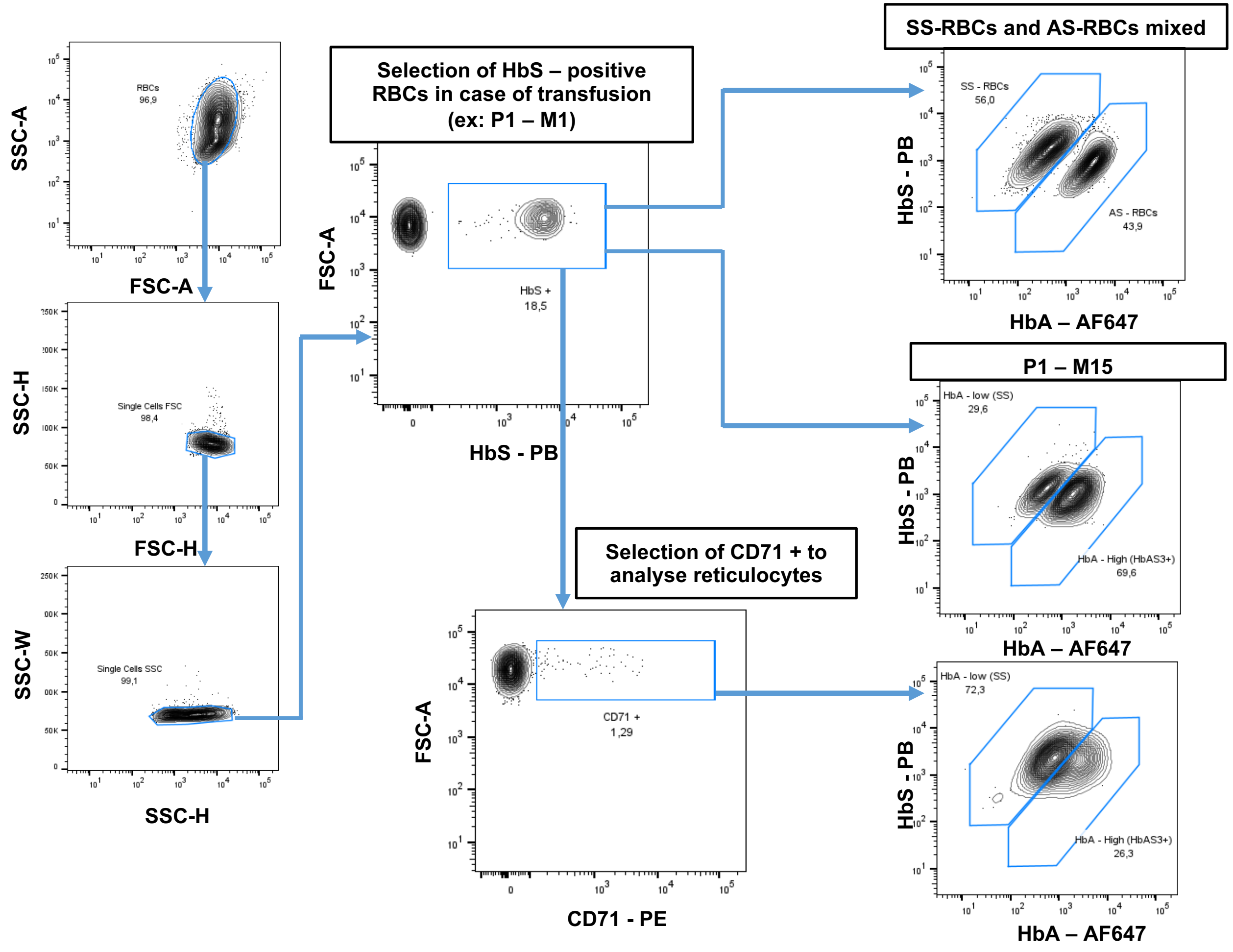

Supplementary Data 3

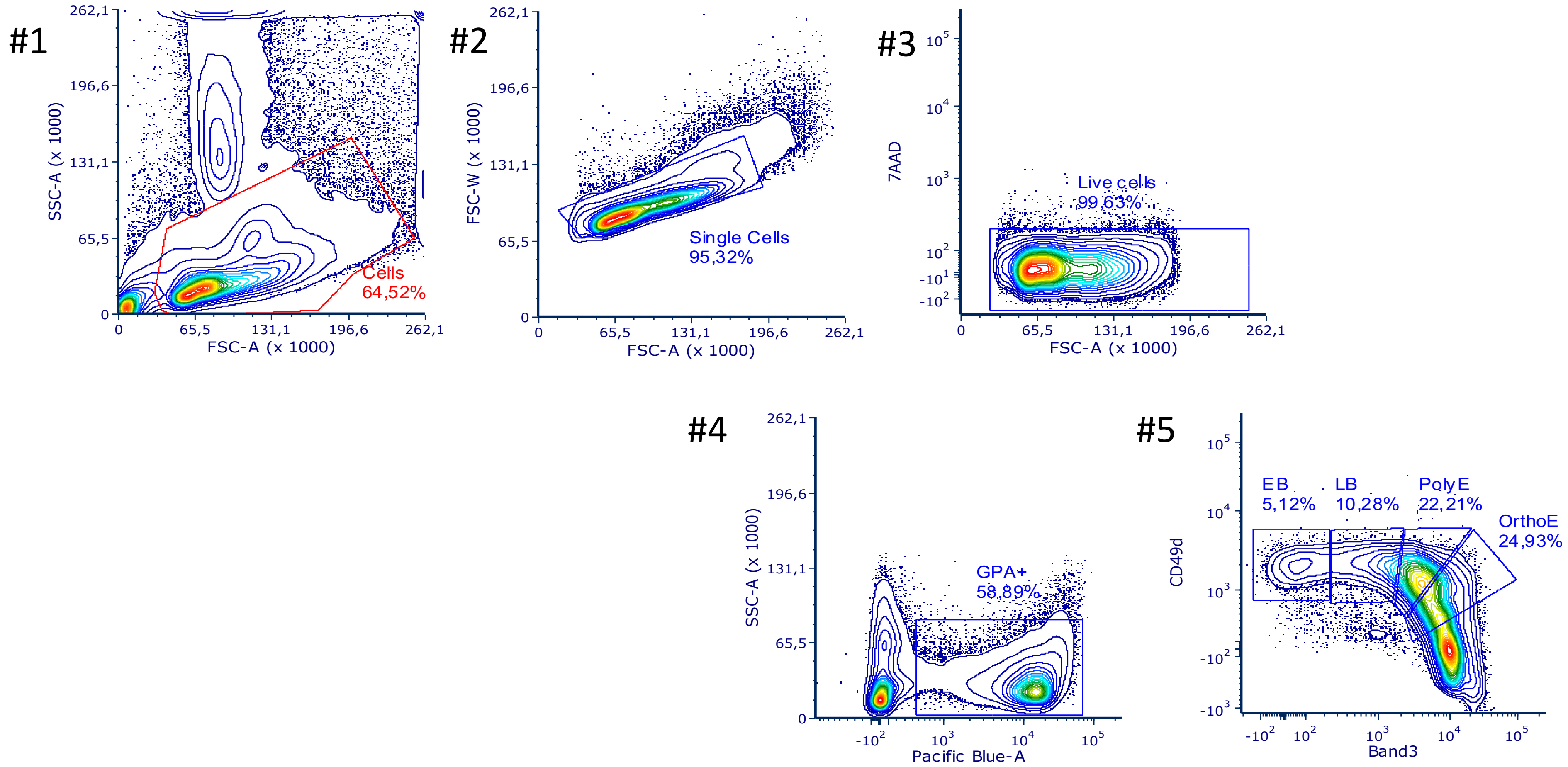

Supplementary Data 4

		cell_8691	cell_7699	cell_16096	cell_10950	cell_9211	cell_2545	cell_6690	cell_12530	cell_12499	cell_2264
	**AllHSC**	**10.6**	**18.2**	11.9	0.0	0.0	0.0	0.0	0.9	2.7	0.0
	**MPP**	4.8	10.3	**13.1**	0.0	0.0	0.0	0.0	10.3	7.0	0.0
	**MLP**	0.0	0.0	0.0	0.1	0.0	0.0	0.0	0.0	0.0	0.9
	**preB**	0.0	0.0	0.0	0.0	5.2	0.0	0.0	0.0	0.0	0.0
	**ImP1**	0.0	0.2	0.0	5.4	0.0	0.0	0.0	0.0	0.0	0.6
	**ImP2**	0.0	0.0	0.0	17.5	0.0	0.5	33.0	0.0	0.0	0.0
NeutroP	**NeutroP0**	0.0	0.0	0.0	8.1	0.0	0.0	0.0	0.0	0.0	1.1
	**NeutroP1**	0.0	0.0	0.0	19.6	0.0	0.0	0.0	0.0	0.0	0.9
	**NeutroP2**	0.0	0.0	0.0	**21.9**	0.0	0.0	0.0	0.0	0.0	0.9
	**NeutroP3**	0.0	0.0	0.0	9.5	8.5	0.0	0.0	0.0	0.0	0.0
	**MonoDCP**	0.0	0.0	0.0	0.0	**68.9**	0.0	0.0	0.0	0.0	0.0
MEP	**MEP1**	0.0	0.0	0.0	4.8	0.0	35.8	59.2	0.0	0.1	0.0
	**MEP2**	0.0	0.0	0.0	0.0	0.0	**97.5**	**79.2**	0.4	1.3	0.0
	**EryP**	0.0	0.0	0.0	0.0	0.0	71.5	63.3	0.0	0.0	0.0
	**MkP**	2.6	0.2	13.1	0.1	0.0	13.5	12.1	**33.5**	**23.8**	0.0
	**EoBasMastP**	0.0	0.0	0.0	0.1	0.5	0.2	0.0	0.0	0.3	0.0
	**Cell types match**	**3**	**2**	**3**	**7**	**3**	**4**	**5**	**2**	**4**	**0**
	**Cell ID**	**HSC**	**HSC**	**MPP**	**NeutroP**	**MonoDCP**	**MEP**	**MEP**	**MkP**	**MkP**	**NA**
