# Supplementary material for: Severe inflammation and lineage skewing are associated with poor engraftment of engineered hematopoietic stem cells in patients with sickle cell disease
Source: Nat Commun. 2025 Apr 1;16:3137. doi: 10.1038/s41467-025-58321-4 (PMC11961595; doi:10.1038/s41467-025-58321-4)
Supplement: Supplementary file 6 — Reporting Summary [file 41467_2025_58321_MOESM6_ESM.pdf]

nature portfolio

Corresponding author(s): Marina Cavazzana

Last updated by author(s): 13/01/25

Reporting Summary

Nature Portfolio wishes to improve the reproducibility of the work that we publish. This form provides structure for consistency and transparency in reporting. For further information on Nature Portfolio policies, see our Editorial Policies and the Editorial Policy Checklist.

Statistics

For all statistical analyses, confirm that the following items are present in the figure legend, table legend, main text, or Methods section.

n/a	Confirmed	
☐	☒	The exact sample size (*n*) for each experimental group/condition, given as a discrete number and unit of measurement
☐	☒	A statement on whether measurements were taken from distinct samples or whether the same sample was measured repeatedly
☐	☒	The statistical test(s) used AND whether they are one- or two-sided<br>*Only common tests should be described solely by name; describe more complex techniques in the Methods section.*
☒	☐	A description of all covariates tested
☐	☒	A description of any assumptions or corrections, such as tests of normality and adjustment for multiple comparisons
☐	☒	A full description of the statistical parameters including central tendency (e.g. means) or other basic estimates (e.g. regression coefficient) AND variation (e.g. standard deviation) or associated estimates of uncertainty (e.g. confidence intervals)
☐	☒	For null hypothesis testing, the test statistic (e.g. *F*, *t*, *r*) with confidence intervals, effect sizes, degrees of freedom and *P* value noted<br>*Give P values as exact values whenever suitable.*
☒	☐	For Bayesian analysis, information on the choice of priors and Markov chain Monte Carlo settings
☒	☐	For hierarchical and complex designs, identification of the appropriate level for tests and full reporting of outcomes
☐	☒	Estimates of effect sizes (e.g. Cohen's *d*, Pearson's *r*), indicating how they were calculated

Our web collection on statistics for biologists contains articles on many of the points above.

Software and code

Policy information about availability of computer code

Data collection	MACSQuant analyzer (Miltenyi Biotec); Spectral Sp6800 and ID7000 (Sony Biotechnologies); Bioanalyzer (Agilent; Fragment Analyzer (Agilent); Xpose spectrophometry (Trinean); AxioObserver Z1 microscope (Zeiss); Illumina NovaSeq 6000 system; SimpliAmp Thermal Cycler (Applied Biosystem); QX200 Droplet Digital PCR System (Biorad); Viia7 Real-Time PCR system (Applied Biosystems); CFX384 Touch Real-Time PCR System (Biorad); NexeraX2 SIL-30AC chromatograph (Shimadzu)
Data analysis	Flowjo software (version 10.8) (FlowJo); LC Solution software (Shimadzu); QuantaSoft software (Biorad); Prism software (version 9; GraphPad); R Studio Software (version 4.0.4; R Core Team); Cell Ranger (version 3.0.2; 10X Genomics); Cell-ID method (Github)

For manuscripts utilizing custom algorithms or software that are central to the research but not yet described in published literature, software must be made available to editors and reviewers. We strongly encourage code deposition in a community repository (e.g. GitHub). See the Nature Portfolio guidelines for submitting code & software for further information.

Data

Policy information about availability of data

All manuscripts must include a data availability statement. This statement should provide the following information, where applicable:

- Accession codes, unique identifiers, or web links for publicly available datasets
- A description of any restrictions on data availability
- For clinical datasets or third party data, please ensure that the statement adheres to our policy

Data Availability
Bulk RNAseq and Single Cell RNAseq data are available at Biostudies EMBL-EBI (S-BSST1524, S-BSST1525, S-BSST1526, S-BSST1257 and S-BSST1258).
Lentiviral integration sites sequencing data are available under NCBI BioProject Accession ID PRJNA1225189.

Code Availability
The full code listing used for the analysis and the figures are available online in the following repository: https://doi.org/10.5281/zenodo.10069444.

Research involving human participants, their data, or biological material

Policy information about studies with human participants or human data. See also policy information about sex, gender (identity/presentation), and sexual orientation and race, ethnicity and racism.

Reporting on sex and gender	Sex and gender are reported in the clinical Table 1
Reporting on race, ethnicity, or other socially relevant groupings	data not collected
Population characteristics	Six homozygous patients with SCD were included in the NCT03964792 GT trial. One patient withdrew from the trial because of persistent hemolytic anemia and the last one withdrawn after inclusion because of the unsatisfying results in the previous treated patients. One pre-screened patient was excluded after detection of suspected clonal hematopoiesis in plerixafor-mobilized HSPCs (a RUNX1 mutation; VAF: 17%). It is noteworthy that P2 was one of the three patients included in the NCT02212535 trial, which demonstrated the safety and efficacy of plerixafor HSPC mobilization; the other two patients in the NCT02212535 trial refused to participate in the GT trial.<br>Prior to GT, all the patients suffered from severe SCD, which was not controlled by strict adherence to supportive treatment (Table 1). In particular, the patients experienced frequent grade 3 vaso-occlusive crises (VOCs) and episodes of life-threatening acute chest syndrome (ACSs) that were not controlled by RBC exchange transfusions and hydroxyurea (HU) treatment (Table 1).
Recruitment	Subjects were selected from SCD followed in Ile de France referree centers. In addition, subjects must meet all inclusion and exclusion criteria. The participation was first proposed to patients included in the NCT02212535 trial, which demonstrated the safety and efficacy of plerixafor HSPC mobilization in SCD patients.
Ethics oversight	The protocol was reviewed by the French Comité de Protection des Personnes and relevant institutional ethics committees.

Note that full information on the approval of the study protocol must also be provided in the manuscript.

Field-specific reporting

Please select the one below that is the best fit for your research. If you are not sure, read the appropriate sections before making your selection.

☒ Life sciences ☐ Behavioural & social sciences ☐ Ecological, evolutionary & environmental sciences

For a reference copy of the document with all sections, see nature.com/documents/nr-reporting-summary-flat.pdf

Life sciences study design

All studies must disclose on these points even when the disclosure is negative.

Sample size	This phase 1/2 study included 4 patients. for the biological study, no sample size calculations were performed in advance.
Data exclusions	No data were excluded from the analysis
Replication	N/A for the clinical study. For the biological study, when possible, samples were from independent donors and experiments were performed with at least 3 biological replicates.
Randomization	Randomization was not performed in this phase 1/2 study including 4 patients

Blinding	Blinding not performed. The analyses were objective measures not subject to bias.

Reporting for specific materials, systems and methods

We require information from authors about some types of materials, experimental systems and methods used in many studies. Here, indicate whether each material, system or method listed is relevant to your study. If you are not sure if a list item applies to your research, read the appropriate section before selecting a response.

Materials & experimental systems

n/a	Involved in the study
☐	☒ Antibodies
☒	☐ Eukaryotic cell lines
☒	☐ Palaeontology and archaeology
☐	☒ Animals and other organisms
☐	☒ Clinical data
☒	☐ Dual use research of concern
☒	☐ Plants

Methods

n/a	Involved in the study
☒	☐ ChIP-seq
☐	☒ Flow cytometry
☐	☒ MRI-based neuroimaging

Antibodies

Antibodies used

Antibodies against the following surface markers: lineage (Lin) custom panel including anti-CD2, CD3, CD4, CD8, CD14, CD15, CD16, CD20, CD56, CD235a (Miltenyi Biotec), CD34 clone 581 (Sony Biotechnologies), CD133 clone 7 (Sony Biotechnologies), CD38 clone HB7 (Sony Biotechnologies), CD90 clone 5E10 (Sony Biotechnologies), CD45RA clone HI100 (Sony Biotechnologies), CD10 clone HI10a (BD Biosciences), CD110 clone BAH1 (BD Biosciences), CD71 clone CY1G4 (Sony Biotechnologies) and CD41 clone HIP8 (Sony Biotechnologies).

Mouse monoclonal antibodies (Ab) against human gamma-globin chain (IQ Products: IQP-363-INT-4), βS-globin chain (Rockland: 200-301-GS5) or βA-globin chain (Rockland: 200-301-GS4). Anti–globin Ab was coupled to phycoerythrin (R-PE) (IQ Products), whereas anti-βS and anti-βA-globin Abs were coupled to Pacific Blue (PB) and to Alexa Fluor 647 (AF-647), respectively, using an Ab conjugation kit (Thermofisher).

mCD45 REA737 (Miltenyi Biotec), hCD45 REA747 (Miltenyi Biotec), CD36 clone CB38 (BD Biosciences), CD71 clone M-A712 (BD Biosciences), CD3 REA613 (Miltenyi Biotec), CD14 clone MφP9(BD Biosciences), CD15 clone VIMC-6 (Miltenyi Biotec), CD19 clone SJ25C1 (BD Biosciences).

Validation

Lin-PE: no validation data are available
CD34 APC-Cy7: https://www.sonybiotechnology.com/us/apc-cy7-anti-human-cd34-9
CD133 PE-Dazzle594: https://www.sonybiotechnology.com/eu/pe-dazzletm-594-anti-human-cd133-1
CD38 BV605: https://www.sonybiotechnology.com/eu/catalog/product/view/id/17911/s/pacific-blue-trade-anti-human-mouse-cd49f-5/category/2/
CD90 PE-Cy5: https://www.sonybiotechnology.com/eu/pe-cy5-anti-human-cd90-thy1-7
CD45RA BV711: https://www.sonybiotechnology.com/eu/brilliant-violet-711-trade-anti-human-cd45ra-7
CD10 APC: https://www.bdbiosciences.com/en-eu/products/reagents/flow-cytometry-reagents/clinical-diagnostics/single-color-antibodies-asr-ivd-ce-ivd/cd10-apc.332777
CD110 BV510: https://www.bdbiosciences.com/en-eu/products/reagents/flow-cytometry-reagents/research-reagents/single-color-antibodies-ruo/bv510-mouse-anti-human-bah-1.743956
CD71 BV650 : https://www.sonybiotechnology.com/eu/brilliant-violet-650tm-anti-human-cd71-1
CD41 BV785: https://www.sonybiotechnology.com/eu/catalog/product/view/id/17969/s/purified-anti-human-cd34-6/category/2/

gamma-globin chain : https://www.iqproducts.nl/products/perinatal/fetal-cell-count-kit-diagnosis-of-fetomaternal-hemorrhage/
βS-globin chain : https://www.rockland.com/categories/primary-antibodies/hemoglobin-beta-s-antibody-200-301-GS5/
βA-globin chain : https://www.rockland.com/categories/primary-antibodies/hemoglobin-a-beta-chain-antibody-200-301-GS4/

mCD45-VioBlue: https://www.miltenyibiotec.com/FR-en/products/cd45-antibody-anti-mouse-reafinity-rea737.html#vioblue:150-ug-in-1-ml
hCD45-APCvio770: https://www.miltenyibiotec.com/FR-en/products/cd45-antibody-anti-human-reafinity-rea747.html#apc-vio-770:100-tests-in-200-ul
CD36-FITC: https://www.bdbiosciences.com/en-eu/products/reagents/flow-cytometry-reagents/research-reagents/single-color-antibodies-ruo/fitc-mouse-anti-human-cd36.555454
CD71-APC: https://www.bdbiosciences.com/en-eu/products/reagents/flow-cytometry-reagents/research-reagents/single-color-antibodies-ruo/apc-mouse-anti-human-cd71.551374
CD3-APC: https://www.miltenyibiotec.com/FR-en/products/cd3-antibody-anti-human-reafinity-rea613.html#apc:100-tests-in-200-ul
CD14-PECy7: https://www.bdbiosciences.com/ko-kr/products/reagents/flow-cytometry-reagents/research-reagents/single-color-antibodies-ruo/pe-cy-7-mouse-anti-human-cd14.562698
CD15-PE: https://www.miltenyibiotec.com/FR-en/products/cd15-antibody-anti-human-vimc6.html#pe:100-tests-in-200-ul
CD19-BV510: https://www.bdbiosciences.com/en-fr/products/reagents/flow-cytometry-reagents/research-reagents/single-color-

antibodies-ruo/bv510-mouse-anti-human-cd19.562947

Animals and other research organisms

Policy information about studies involving animals; ARRIVE guidelines recommended for reporting animal research, and Sex and Gender in Research

Laboratory animals	Non-obese diabetic severe combined immunodeficiency gamma (NSG) mice (NOD.CgPrkdcscid Il2rgtm1Wjl/SzJ, Charles River Laboratories, St Germain sur l'Arbresle, France)
Wild animals	The study did not involve wild animals.
Reporting on sex	We have transplant HSPCs exclusively to immunodeficient female mice because females support better engraftment of human cells compared to male mice
Field-collected samples	The study did not involve samples collected from the field.
Ethics oversight	All experiments and procedures were performed in compliance with the French Ministry of Agriculture's regulations on animal experiments and were approved by the regional Animal Care and Use Committee (APAFIS#2101-2015090411495178 v4).

Note that full information on the approval of the study protocol must also be provided in the manuscript.

Clinical data

Policy information about clinical studies

All manuscripts should comply with the ICMJE guidelines for publication of clinical research and a completed CONSORT checklist must be included with all submissions.

Clinical trial registration	NCT03964792
Study protocol	The Clinical Study Protocol is available on ClinicalTrials.gov under the clinical trial registration numbers listed above.
Data collection	A detailed Schedule of Events is located in the Clinical Study Protocol for the Study NCT03964792 and in the Table 1 and Extended Data Table 3 in the text.
Outcomes	All study outcomes are defined in the Clinical Study Protocol located on clinicaltrials.gov under clinical trial registration number NCT03964792

Plants

Seed stocks	NA
Novel plant genotypes	*Describe the methods by which all novel plant genotypes were produced. This includes those generated by transgenic approaches, gene editing, chemical/radiation-based mutagenesis and hybridization. For transgenic lines, describe the transformation method, the number of independent lines analyzed and the generation upon which experiments were performed. For gene-edited lines, describe the editor used, the endogenous sequence targeted for editing, the targeting guide RNA sequence (if applicable) and how the editor was applied.*
Authentication	*Describe any authentication procedures for each seed stock used or novel genotype generated. Describe any experiments used to assess the effect of a mutation and, where applicable, how potential secondary effects (e.g. second site T-DNA insertions, mosiacism, off-target gene editing) were examined.*

Flow Cytometry

Plots

Confirm that:

☒ The axis labels state the marker and fluorochrome used (e.g. CD4-FITC).

☒ The axis scales are clearly visible. Include numbers along axes only for bottom left plot of group (a 'group' is an analysis of identical markers).

☒ All plots are contour plots with outliers or pseudocolor plots.

☒ A numerical value for number of cells or percentage (with statistics) is provided.

Methodology

Sample preparation	In line with the trial protocol, MPB was collected for IMP manufacturing and peripheral blood was sampled regularly during the follow-up period. Mononuclear cells were isolated from MPB using standard Ficoll density gradient separation. The

absolute lymphocyte count was determined using Trucount Tubes (BD Bioscience). CD34+. cells were immunoselected using the CliniMACS system (Miltenyi Biotec). Human HSPC were stained and fixed before analysis.

Murine cells (bone marrow) were analyzed for chimerism (mouse and human CD45 expression) and lineage specific markers (CD3, CD14, CD15, CD19, CD36, CD71). Bone marrow was flushed from femur and tibia and passed through a cell strainer to obtain a single cell suspension before staining.

Instrument	MACSQuant analyzer (Miltenyi Biotec); Spectral Sp6800 or ID7000 (Sony Biotechnologies)
Software	Flowjo software (version 10.8) (FlowJo)
Cell population abundance	>75% of the cells in patient and HD HSPC samples were CD34+ and the analysis was performed gating only on the CD34 positive cells
Gating strategy	Linear FSC-A versus linear SSC-A was used to gate the human HSPC. Within the human cells, FSC-A versus FSC-H was used to gate on single cells. In the single cell population, FSC-A versus Live Dead blue was used to gate on the Live Dead blue negative (live) cells. Within the live cells, CD34 versus Lineage was used to gate on CD34+Lin-, then the CD133 vs CD38 to gate on CD133+CD38- and the CD90 vs CD45RA to gate on CD90+CD45RA- to determine the HSC subpopulation. Within the CD133-CD38+ cells, the CD10 vs CD45RA was used to identify the CD10+ (BNKP). In CD10- , the CD110 vs CD45RA was used to determine the MEP (CD110+CD45RA-), GMP (CD110-CD45RA+) and the CMP (CD110-CD45RA-).<br><br>Linear FSC-A versus linear SSC-A was used to gate the murine cells (bone marrow). Within the murine cells, FSC-A versus FSC-H was used to gate on single cells. In the single cell population, FSC-A versus 7AAD was used to gate on the 7AAD negative (live) cells. Within the live cells, hCD45-APCvio770 versus mCD45-VioBlue, was used to gate on the hCD45-APCvio770 and mCD45-VioBlue positive cells. Within the hCD45-APCvio770 and mCD45-VioBlue positive cells, FSC-A versus hCD45-APCvio770 was used to gate on the hCD45-APCvio770 positive cells. Within the hCD45-APCvio770 positive cells, FSC-A versus lineage specific markers (CD3, CD14, CD15, CD19, CD36, CD71), was used to gate on the positive cells.

☒ Tick this box to confirm that a figure exemplifying the gating strategy is provided in the Supplementary Information.

Magnetic resonance imaging

Experimental design

Design type	NA
Design specifications	*Specify the number of blocks, trials or experimental units per session and/or subject, and specify the length of each trial or block (if trials are blocked) and interval between trials.*
Behavioral performance measures	*State number and/or type of variables recorded (e.g. correct button press, response time) and what statistics were used to establish that the subjects were performing the task as expected (e.g. mean, range, and/or standard deviation across subjects).*

Acquisition

Imaging type(s)	*Specify: functional, structural, diffusion, perfusion.*
Field strength	*Specify in Tesla*
Sequence & imaging parameters	*Specify the pulse sequence type (gradient echo, spin echo, etc.), imaging type (EPI, spiral, etc.), field of view, matrix size, slice thickness, orientation and TE/TR/flip angle.*
Area of acquisition	*State whether a whole brain scan was used OR define the area of acquisition, describing how the region was determined.*

Diffusion MRI ☐ Used ☐ Not used

Preprocessing

Preprocessing software	*Provide detail on software version and revision number and on specific parameters (model/functions, brain extraction, segmentation, smoothing kernel size, etc.).*
Normalization	*If data were normalized/standardized, describe the approach(es): specify linear or non-linear and define image types used for transformation OR indicate that data were not normalized and explain rationale for lack of normalization.*
Normalization template	*Describe the template used for normalization/transformation, specifying subject space or group standardized space (e.g. original Talairach, MNI305, ICBM152) OR indicate that the data were not normalized.*
Noise and artifact removal	*Describe your procedure(s) for artifact and structured noise removal, specifying motion parameters, tissue signals and physiological signals (heart rate, respiration).*

Volume censoring | *Define your software and/or method and criteria for volume censoring, and state the extent of such censoring.*

Statistical modeling & inference

Model type and settings | *Specify type (mass univariate, multivariate, RSA, predictive, etc.) and describe essential details of the model at the first and second levels (e.g. fixed, random or mixed effects; drift or auto-correlation).*

Effect(s) tested | *Define precise effect in terms of the task or stimulus conditions instead of psychological concepts and indicate whether ANOVA or factorial designs were used.*

Specify type of analysis: ☐ Whole brain ☐ ROI-based ☐ Both

Statistic type for inference | *Specify voxel-wise or cluster-wise and report all relevant parameters for cluster-wise methods.*

(See Eklund et al. 2016)

Correction | *Describe the type of correction and how it is obtained for multiple comparisons (e.g. FWE, FDR, permutation or Monte Carlo).*

Models & analysis

n/a	Involved in the study
☐	☐ Functional and/or effective connectivity
☐	☐ Graph analysis
☐	☐ Multivariate modeling or predictive analysis

Functional and/or effective connectivity | *Report the measures of dependence used and the model details (e.g. Pearson correlation, partial correlation, mutual information).*

Graph analysis | *Report the dependent variable and connectivity measure, specifying weighted graph or binarized graph, subject- or group-level, and the global and/or node summaries used (e.g. clustering coefficient, efficiency, etc.).*

Multivariate modeling and predictive analysis | *Specify independent variables, features extraction and dimension reduction, model, training and evaluation metrics.*
